# Determinants of inequalities in years with disability: an international-comparative study

**DOI:** 10.1093/eurpub/ckaa194

**Published:** 2020-11-22

**Authors:** Wilma J. Nusselder, José Rubio Valverde, Matthias Bopp, Henrik Brønnum-Hansen, Patrick Deboosere, Ramune Kalediene, Katalin Kovács, Mall Leinsalu, Pekka Martikainen, Gwenn Menvielle, Enrique Regidor, Bodgan Wojtyniak, Johan P. Mackenbach

**Affiliations:** 1 Department of Public Health, Erasmus MC, University Medical Center Rotterdam, Rotterdam, the Netherlands; 2 Epidemiology, Biostatistics and Prevention Institute, University of Zürich, Zürich, Switzerland; 3 Department of Public Health, Faculty of Health Sciences, University of Copenhagen, Copenhagen, Denmark; 4 Department of Sociology, Vrije Universiteit Brussel, Brussels, Belgium; 5 Department of Health Management. Lithuanian University of Health Sciences, Kaunas, Lithuania; 6 Aging and health. Demographic Research Institute, Budapest, Hungary; 7 Department of Sociology. Stockholm Centre for Health and Social Change, Södertörn University, Stockholm, Sweden; 8 Department of Epidemiology and Biostatistics, National Institute for Health Development, Tallinn, Estonia; 9 Population Research Unit, Faculty of Social Sciences, University of Helsinki, Helsinki, Finland; 10 Sorbonne Université, INSERM, Institut Pierre Louis d’Epidémiologie et de Santé Publique (IPLESP UMRS 1136), Paris, France; 11 Department of Public Health & Maternal and Child Health, Faculty of Medicine, Universidad Complutense de Madrid, Madrid, Spain; 12 CCIBER Epidemiología y Salud Pública, Instituto Salud Carlos III, Madrid, Spain; 13 Department of Population Health Monitoring and Analysis. National Institute of Public Health-National Institute of Hygiene, Warsaw, Poland

## Abstract

**Background:**

Persons with a lower socioeconomic position spend more years with disability, despite their shorter life expectancy, but it is unknown what the important determinants are. This study aimed to quantify the contribution to educational inequalities in years with disability of eight risk factors: father’s manual occupation, low income, few social contacts, smoking, high alcohol consumption, high body-weight, low physical exercise and low fruit and vegetable consumption.

**Methods:**

We collected register-based mortality and survey-based disability and risk factor data from 15 European countries covering the period 2010–14 for most countries. We calculated years with disability between the ages of 35 and 80 by education and gender using the Sullivan method, and determined the hypothetical effect of changing the prevalence of each risk factor to the prevalence observed among high educated (‘upward levelling scenario’), using Population Attributable Fractions.

**Results:**

Years with disability among low educated were higher than among high educated, with a difference of 4.9 years among men and 5.5 years among women for all countries combined. Most risk factors were more prevalent among low educated. We found the largest contributions to inequalities in years with disability for low income (men: 1.0 year; women: 1.4 year), high body-weight (men: 0.6 year; women: 1.2 year) and father’s manual occupation (men: 0.7 year; women: 0.9 year), but contributions differed by country. The contribution of smoking was relatively small.

**Conclusions:**

Disadvantages in material circumstances (low income), circumstances during childhood (father’s manual occupation) and high body-weight contribute to inequalities in years with disability.

**Key points**

- Between the age of 35 and 80, on average low educated men and women spend 4.9 and 5.5 years more with disability than their high educated peers.
- The three most important contributors to educational inequalities in years with disability were low income, high body-weight and father’s occupation.
- The contribution of smoking was relatively small reflecting high excess mortality associated with smoking.

## Introduction

Persons with low levels of education spend more years with disability despite their shorter life expectancy than persons with higher levels of education.^1^^,^^2^ This is the net result of two opposing effects: (i) more years with disability at the expense of fewer years free of disability and (ii) fewer years with disability at the expense of years lost to mortality.^3^ Years with disability impose an additional societal challenge to aging populations, because persons with disability are hospitalized more often, need more medical and long-term care and participate less in (paid) work.

In an influential article on compression of morbidity, James Fries^4^ postulated that avoiding smoking, physically inactivity and an unhealthy diet would reduce the number of years with disability. Most unhealthy behaviours are more frequent among persons with a lower education.^5^ Also other factors, including poor housing conditions, financial difficulty during childhood, financial strain, lack of a social network and adverse working conditions are associated with higher disability and the prevalence of these factors is higher among persons with a lower education.^5^

A recent study examined the contribution of several of these risk factors to educational inequalities in life expectancy, showing the largest contribution of smoking, followed by high body-weight, and low income.^6^ It is unclear whether the same factors contribute similarly to inequalities in years with disability, given opposite effects of mortality and disability on this outcome.^7^ Prior studies showed that obesity^8^^,^^9^ and lack of physical activity^10^ are associated with more years with disability, but for smoking the results are mixed.^11^ No prior study did examine the contribution of risk factors on educational inequalities in years with disability.

This study aims to estimate the contribution of a broad range of risk factors for mortality and disability, which have been shown to be differentially distributed between low and high educated, to inequalities in years with disability in Europe. We will include eight risk factors: father’s manual occupation as an indicator of conditions in which persons have grown up,^12^^,^^13^ low income as an indicator of current material living conditions,^14^ few social contacts as an indicator of psychosocial conditions^15^ and five behavioural risk factors, namely smoking,^16^ high alcohol consumption,^17^ high body-weight,^18^ low physical activity^19^ and low fruit and vegetable consumption.^20^ Together, these risk factors cover different but overlapping explanatory perspectives. Behavioural risk factors can be conceptualized as being ‘downstream’ in the causal pathway between level of education and mortality. Father’s manual occupation and low income partly determine that lower and higher educated people have different health-related behaviours, and should therefore be seen as more ‘upstream’ than health-related behaviours.^21^ Father’s manual occupation partly determines a person’s educational achievement,^22^ and should therefore not be seen as a possible mediator but as a factor capturing the persistent effect of childhood conditions on the risks of mortality and disability in later life. For persons aged 35–80 in 15 European countries, we evaluate the hypothetical effect of an ‘upward levelling’ scenario, where exposure to each of these risk factors in all educational groups is set to the prevalence observed among high educated.

## Methods

### Data

We used harmonized register-based mortality data by age, gender and education from 15 European countries: Finland, Sweden, Norway, Denmark (in the North of Europe), England and Wales, the Netherlands, Belgium, Austria, Switzerland (in the West of Europe), France, Spain (in the South of Europe), Hungary, Poland, Lithuania and Estonia (in the East of Europe). These data covered complete national populations, except for England and Wales and France (1% representative samples), and the Netherlands (65% of population covered). We used data for the period around 2010–14, except for Sweden (2005–08), Norway (2006–09) and France (2004–07). Most data stemmed from longitudinal mortality follow-up, except for Hungary and Poland where cross-sectional unlinked studies were used. Supplementary appendix S1 provides details about the mortality data sources.

Data on disability and income were obtained from the European Union Statistics on Income and Living Conditions (EU-SILC) survey. Data on other risk factors were obtained from round 7 (2014) of the European Social Survey. This survey was designed to collect harmonized data on inequalities in risk factors, but did not include data on income for all countries.^23^Supplementary appendix S2 gives more information on these surveys. For EU-SILC, we pooled years 2010 and 2014, except for Sweden, Norway and France, where we used 2005 and 2009 to better match with the mortality data. Because EU-SILC is a rotating panel survey, we did not include intermediate years to avoid including respondents multiple times. To assess disability, we used the Global Activity Limitation Indicator (GALI). GALI is an indicator of participation restriction, which refers to limitation in the performance of roles and social involvement in activities such as work, leisure, parenting, housework and social life.^24^ It was based on the question ‘For at least the past 6 months, to what extent have you been limited because of a health problem in activities people usually do?’ Respondents were classified as having a disability if they responded with ‘Yes, severely’ or ‘Yes, to some extent’. GALI is a validated and relatively accurate indicator to assess disability,^25–29^ although there are some variations in wording between countries. More information on the GALI indicators is given in the References.^24^^,^^29–31^

We used the following criteria for the selection of risk factors, in addition to whether the prevalence of the risk factor has been reported to differ between educational groups: (i) an estimate of the relative risk of mortality and disability is available in the literature and (ii) estimates of the prevalence of the risk factor by level of education in the 15 countries are available from internationally harmonized surveys. These criteria resulted in the selection of eight risk factors**:** father’s manual occupation, low income, few social contacts, smoking, high alcohol consumption, high body-weight, low physical activity and low fruit and vegetable consumption. We could not include housing and working conditions, because of the absence of relative risks of mortality and disability for these risk factors as measured in the surveys. Supplementary appendix S3 provides more information about the risk factors.

The highest completed level of education was used as indicator of socioeconomic position. Education is usually determined early in life, therefore better avoiding problems of reverse causation than other SEP measures^32^ and mortality data by education are available for more countries than data by other SEP-indicators. Level of education was categorized into three levels: low (ISCED 0–2), medium (ISCED 3–4) and high (ISCED 5–6). In the presentation of the results, we focussed on inequalities between low and high education.

Relative risks were preferably based on meta-analyses or reviews, and if not available, we pooled results from individual studies. If more relative risks were presented in the literature, we selected relative risks adjusted for age, gender and other risk factors that were not on the causal pathway between the risk factor and mortality/disability, and for adult socioeconomic position. For the mortality, relative risks were highest for smoking (RR of 2.2), obesity (RR of 1.7) and high alcohol consumption (RR of 1.4). For disability relative risks were highest for obesity (RR of 1.8), low physical activity, low income (both RR of 1.5) and overweight (RR of 1.4). For more information about the relative risks of mortality and disability, see Supplementary appendix S4.

## Analyses

For each country, we estimated the contribution of each risk factor to the inequality in years with disability by comparing years with disability in the current situation (baseline scenario) with the counterfactual situation where the prevalence of the risk factor of low educated was set to the observed level of high educated (**‘**upward levelling scenario’). The analyses were conducted for men and women separately and restricted to ages 35–80 years, because mortality data were not available for all countries below age 35 and education becomes less reliable at higher ages.

First, we calculated for each educational group mortality rates, disability prevalence and risk factor prevalence, by age for the baseline scenario (no change). Restricted cubic spline models were used to smooth the age- and education-specific prevalences.

Second, we calculated for each educational group Population Attributable Fractions (PAFs) for mortality and disability, using risk factor prevalence in the *i*th exposure category (*P_i_*), the prevalence in the same category according to the upward levelling scenario (*P*’_*i*_) and relative risks (RR_*i*_) for mortality and disability, respectively, for each of the *n* categories of the risk factor.^33^

$$\mathrm{PAF}=\;\frac{\sum_{i=1}^{n}P_{i}{\mathrm{RR}}_{i}-\;\sum_{i=1}^{n}{P'}_{i}{\mathrm{RR}}_{i}}{\sum_{i=1}^{n}P_{i}{\mathrm{RR}}_{i}}.$$

Third, we calculated for each educational group mortality rates and disability prevalence by age for the upward levelling scenario by applying the PAFs to the baseline mortality rates and disability prevalence.

Fourth, we calculated for each educational group years with disability for the baseline and upward levelling scenario using the Sullivan method^34^ with the age-specific mortality rates and disability prevalence as input. According to this method, person years at each age are split into years with and without disability by using the prevalence of disability.

Finally, we calculated the difference in years with disability between the baseline and upward levelling scenario. Because equalizing the distribution of risk factors does not affect high educated, the difference for low educated equals the reduction in inequality between low and high educated.

In all graphs and tables, we present a European average, calculated as population-weighted average of the values for each country in our study. All 95% confidence intervals (95% CIs) were determined with bootstrapping (1000 samples), taking into account uncertainty regarding mortality, disability and risk factors.

## Results

### Inequalities in years with disability and prevalence of risk factors

Years with disability between the ages of 35 and 80 vary by country, gender and educational level (table 1 and Supplementary appendix S5). For all countries combined, low educated men spend 12.3 (95% CI 12.1–12.6) years with disability, varying between 9.5 (95% CI 8.3–10.4) years in Sweden and 19.1 (95% CI 17.9–20.2) years in Austria. High educated men spend fewer years with disability, namely 7.4 (95% CI 7.1–7.6) years for the European average and varying between 3.8 (95% CI 3.2–4.5) years in Norway and 12.6 (95% CI 11.8–13.5) years in Estonia. The inequalities in years with disability between low and high educated men are on average 4.9 (95% CI 4.7–5.2) years, varying between 2.0 (95% CI 1.0–3.6) years in Denmark and 8.2 (95% CI 7.0–9.4) years in Austria. For all countries combined, low educated women spend 15.0 (95% CI 14.7–15.2) years with disability, varying between 13.6 (95% CI 13.1–14.0) years in France and 20.7 (95% CI 19.5–22.7) years in Estonia. High educated women spend on average 9.4 years with disability between the ages of 35 and 80 (95% CI 9.2–9.7), ranging between 7.6 (95% CI 6.7–8.5) years in Norway and 13.7 (95% CI 13.1–14.4) years in Finland. The inequality in years with disability between low and high educated women is 5.5 (95% CI 5.3–5.7) years difference in favour of high educated, ranging between 1.2 (95% CI 0.0–2.9) year for Denmark and 9.3 (95% CI 8.7–9.9) years for Hungary.

**Table 1:** Educational inequalities in years with disability between ages 35 and 80 (in years)

	Years with disability			**Inequality** ^a^ between low and high educated (95% CI)	
	Low	Med	High		
Men					
North					
Finland	13.9	12.9	9.9	4.0	(3.0, 5.0)
Sweden	9.5	8.0	4.9	4.6	(3.7, 5.8)
Norway	10.4	7.9	3.8	6.6	(5.3, 7.7)
Denmark	11.4	10.7	9.4	2.0	(1.0, 3.6)
West					
England/Wales	13.0	9.7	7.5	5.5	(4.8, 6.1)
the Netherlands	13.4	11.2	8.9	4.5	(3.3, 5.5)
Belgium	13.6	8.9	6.2	7.3	(6.6, 8.2)
Austria	19.1	14.0	10.9	8.2	(7.0, 9.4)
Switzerland	13.4	10.2	8.1	5.3	(3.4, 7.2)
South					
France	11.2	9.3	6.3	4.9	(4.3, 5.4)
Spain	11.2	9.0	7.0	4.1	(3.9, 4.4)
East					
Hungary	13.8	11.9	8.6	5.2	(4.5, 5.8)
Poland	12.2	10.8	8.0	4.2	(3.5, 5.0)
Lithuania	11.9	9.1	6.9	5.0	(3.4, 6.7)
Estonia	16.2	13.9	12.6	3.7	(2.4, 4.6)
European mean^b^	12.3	9.9	7.4	4.9	(4.7, 5.2)
Women					
North					
Finland	16.2	16.4	13.7	2.5	(1.4, 3.8)
Sweden	14.0	11.3	8.8	5.1	(3.2, 6.6)
Norway	14.8	10.2	7.6	7.2	(5.8, 8.5)
Denmark	13.7	13.1	12.6	1.2	(0.0, 2.9)
West					
England/Wales	14.4	10.5	9.2	5.2	(4.5, 5.8)
the Netherlands	17.5	14.9	11.5	6.0	(5.1, 7.1)
Belgium	16.8	12.0	9.0	7.9	(7.2, 8.7)
Austria	19.3	14.7	12.5	6.8	(6.0, 7.7)
Switzerland	13.8	12.0	12.4	1.4	(0.2, 2.7)
South					
France	13.6	10.8	8.5	5.1	(4.8, 5.7)
Spain	14.5	10.0	8.1	6.4	(6.1, 6.6)
East					
Hungary	18.8	13.3	9.5	9.3	(8.7, 9.9)
Poland	15.2	12.8	10.2	5.1	(4.1, 5.6)
Lithuania	16.6	13.3	7.8	8.8	(6.1, 10.7)
Estonia	20.7	17.6	12.9	7.8	(5.8, 9.0)
European mean^b^	15.0	11.6	9.4	5.5	(5.3, 5.8)

a Low-high.

b Population-weighted means of all European countries in the analysis.

**Figure 1:**
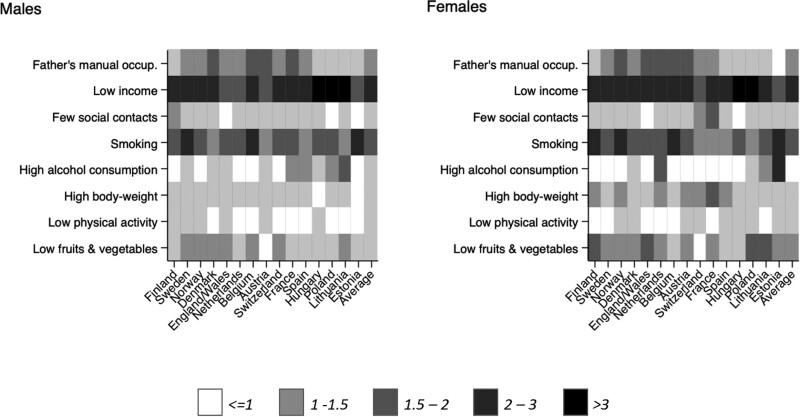
Educational inequalities in risk factors expressed as prevalence ratio, ages 35–79 years, by country and by gender Note: Prevalence ratio >1 indicates a higher prevalence among low educated. Average = population-weighted average of all European countries in the analysis.

Educational inequalities in prevalence of the risk factors are shown in figure 1 (more extensive data and figures in colour are shown in Supplementary appendix S6). Most risk factors are more prevalent among low than among high educated, but there are some exceptions, e.g. with small or opposite gradients for high alcohol consumption and low physical activity. The largest inequalities are found for low income (with prevalence ratios often exceeding 3.0) and smoking.

### Estimated effect of ‘upward levelling’

**Figure 2:**
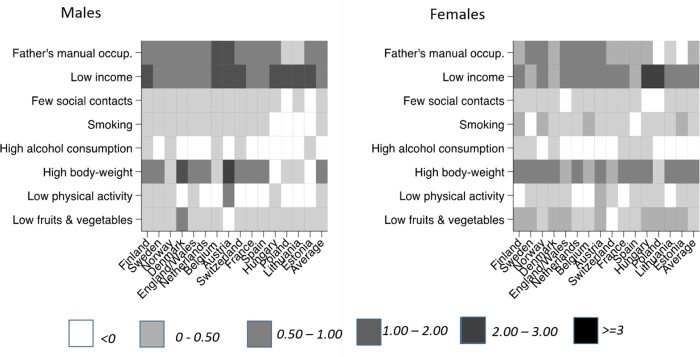
Effect of ‘upward levelling’ of risk factor prevalence on years with disability between age 35 and 80 years, by country and by gender

Table 2 (average of all countries), figure 2 and Supplementary appendix S7 (both by country) present the absolute change in years with disability among low educated obtained through changing their exposure to that of high educated (‘upward levelling scenario’). For all countries combined, among men the largest change is seen for low income (1.00 year; 95% CI 0.96–1.05). Upward levelling of low income thus reduces years with disability among low educated men with 1.0 year. It also reduces the inequality between low and high educated men with 1.0 year, because high educated are unaffected by the upward levelling scenario. After income, father’s manual occupation (0.70 year; 95% CI 0.68–0.71) and high body-weight (0.64 year; 95% CI 0.44–0.81) contribute most. For women, the largest effect is seen for low income (1.42 year; 95% CI 1.36–1.49), followed by high body-weight (1.19 years; 95% CI 0.83–1.48) and father’s manual occupation (0.86 year; 95% CI 0.84–0.88). The effects for the other risk factors are <0.5 year.

**Table 2:** Years with disability (YwD) between age 35 and 80 and effects of upward levelling, European average, by gender

	Men		Women	
European average	YwD	95% CI	YwD	95% CI
Low	12.32	(12.05, 12.55)	14.96	(14.73, 15.20)
Medium	9.90	(9.90, 10.11)	11.57	(11.26, 11.74)
High	7.39	(7.12, 7.61)	9.42	(9.15, 9.72)
Differences low–high	4.92	(4.69–5.17)	5.48	(5.30, 5.71)

Upward levelling scenario	Change in difference low–high = change in DLE low			
European average	YwD	95% CI	YwD	95% CI

Father manual occupation	0.70	(0.68, 0.71)	0.86	(0.84, 0.88)
Low income	1.00	(0.96, 1.05)	1.42	(1.36, 1.49)
Few social contacts	0.01	(−0.01, 0.03)	0.04	(0.02, 0.07)
Smoking	0.17	(0.10, 0.21)	0.19	(0.08, 0.31)
High alcohol	−0.01	(−0.02, −0.01)	0.00	(0.00, 0.00)
High body-weight	0.64	(0.44, 0.81)	1.19	(0.83, 1.48)
Low physical activity	0.05	(−0.01, 0.11)	0.10	(−0.11, 0.22)
Low fruit and vegetables	0.25	(0.17, 0.32)	0.45	(0.31, 0.55)

For most countries, similar to the European average, upward levelling of low income shows the largest effect. The effects for low income are larger in the East of Europe, particularly for women exceeding 2 years in Hungary and Poland, and smaller in Sweden, Denmark and Norway (men). In some countries, high body-weight shows larger effects of upward levelling than low income, for instance Sweden, Denmark, Austria (men), France (women), Spain (women) and the Netherlands (women).

## Discussion

Between the age of 35 and 80 for all countries combined, low educated men and women spend 4.9 and 5.5 years more with disability than their high educated peers. This study assessed the contribution of 8 risk factors to these inequalities, showing largest contributions for low income (for all countries combined: 1.0 year in men and 1.4 year in women), high body-weight (0.6 and 1.2 years) and father’s manual occupation (0.7 and 0.9 years), but contributions differed by country. The effect of smoking is relatively small (0.17 and 0.19).

Risk factors contributing the most to inequalities in years with disability only partly overlap with those contributing the most to inequalities in life expectancy.^6^ Smoking was the most important contributor to inequalities in life expectancy, followed by low income and high body-weight. Our study showed that smoking contributed little to inequalities in years with disability. We found larger contributions of father’s manual occupation, low income and high body-weight than for smoking. These factors are more strongly associated with disability than with mortality, as is reflected in the relative risks derived from literature.

For a large contribution to inequalities in years with disability, the prevalence of the risk factor should differ substantially between high and low educated, the risk factor should substantially increase the risk of disability, and this increase should be larger than for mortality. Low income and smoking are both characterized by large inequalities in prevalence between low and high educated. The combination of large inequalities in prevalence and the stronger association with disability than with mortality yields large contributions of low income to inequalities in years with disability. The contributions are particularly large in the East of Europe, where inequalities in prevalence of low income are large (figure 1 and Supplementary table S6). High body-weight and father’s manual occupation share these characteristics, but in general to a lesser extent. That smoking contributes little to inequalities in years with disability is mainly because its strong association with mortality. Mortality largely cancels out the opposite effect of increased disability associated with smoking. This strong association with mortality explains the large contribution of smoking to inequalities in life expectancy.

That some risk factors have a larger effect on mortality and others on disability can be largely traced back to different diseases that are associated with the risk factors, as well as their interaction with environmental and personal factors.^35^ Smoking is known to increase the risk of several fatal conditions, such as several cancers, e.g. lung cancer, cardiovascular diseases and respiratory diseases. These diseases contribute largely to mortality but less to disability.^36^ High body-weight increases the risks of musculoskeletal and mental diseases. Low income increases the risk of mental diseases. These diseases contribute largely to disability but less to mortality.^36^ Low income may additionally impact on disability by reducing access to (rehabilitative) care relevant for disability. Depending on the organization of the health care system and health insurance, care related to fatal conditions may require less out-of-pocket payments than care related to disability.

To our knowledge, there are no prior studies that examined the contribution of risk factors to educational differences in years with disability. One study^5^ assessed the contribution of several risk factors to inequalities in the prevalence of GALI disability, and showed the important contribution of work-related factors (which could not be included in our study), in addition to behavioural and living conditions. Our study adds that some behavioural factors, such as high body-weight, also contribute to inequalities in years with disability, but the contribution of smoking is small. Whether occupational factors contribute substantially to years with disability needs to be examined in further research that has information on both exposure to these occupational factors and their relative risks of mortality and disability.

### Strengths and limitations

The strength of this study is that we include a broad range of risk factors and take into account the effects of risk factors on both disability and mortality to assess their net contribution to inequalities in years with disability. The PAF approach allowed combining risk factor exposure data from harmonized European surveys with relative risks from the literature where possible using meta-analyses or reviews. Obtaining comparable estimates of the contribution to inequalities in years with disability from longitudinal studies for several countries is generally not possible, as they often lack the power to allow stratification by SEP, their follow-up is too short and multi-country cohort studies are scarce. However, the PAF approach has limitations. First, we could not include housing and working conditions because the PAF method requires that risk factor exposure is defined and categorized similar for the relative risks and the prevalence data. Second, for disability we were not able to use relative risks based on reviews or meta-analyses due to unavailability of studies, therefore, we averaged relative risks from individual studies if needed. For mortality, we could generally use relative risks based on reviews or meta-analyses. Third, the relative risks of disability were based on different disability measures and not on the GALI indicator. Relative risks based on the GALI indicator were not available, which is a limitation. However, prior research showed that the GALI indicator is strongly associated with other disability measures^25^^,^^28^^,^^31^ used in this study. Fourth, we used the same relative risks for all countries, education groups, ages and for both genders. One set of relative risks for all countries may be more problematic for disability than for mortality, as disability arises from the interplay between impaired health and the environment.^35^ It may also be more problematic for low income than for behavioural risk factors. We expect that social security systems in different countries buffer the impact of poverty on disability and mortality to a different extend, and less in Eastern Europe. Using the same relative risks for all educational groups ignores that e.g. smoking is associated with higher mortality for lower educated people than for higher educated people.^37^ These biases are likely to affect the relative risks of both mortality and disability in the same direction, the net effect on inequalities in years with disability will be partly cancelled out.

Other limitations are that no statements can be made regarding the duration of exposure, due to the cross-sectional assessment of exposure in the surveys, Also, we could not take into account lag-times before mortality and disability effects can be expected to occur. Also, we analyzed the risk factor contributions one-by-one. Since ‘downstream’ risk factors (i.e. high body-weight, smoking, physical activity) are determined in part by ‘upstream’ factors like income, or by other downstream factors (i.e. high body-weight by diet and physical activity,) the contribution of all risk factors together may be less than the sum of the individual contributions, but is expected to be larger than each of the contributions. Finally, cultural differences, differences in reporting behaviour, differences in exact wording of survey questions and differences in survey participation and survey years may hamper comparability between countries. The variation in the results between countries must therefore be interpreted with caution.

The broader perspective applied in this study takes into account effects of risk factors through mortality and disability and adds risk factors beyond the best documented behavioural factors. The contributions of the factors to inequalities in years with disability should be seen as indications of the order of magnitude.

## Conclusion and implications

Disadvantages in material circumstances (low income), circumstances in which children grow up (father’s manual occupation) and high body-weight contributed to more years with disability among low educated. Smoking hardly contributed to the inequality in years with disability because higher mortality associated with smoking reduced the time alive with disability.

Our findings suggest that policies with the aim to tackle inequalities in years with disability should focus on reducing inequalities in low income (e.g. through progressive income taxation), in high body-weight (e.g. through health promotion programmes) and in unfavourable circumstances during childhood (e.g. through family support programmes).

## Supplementary data

Supplementary data are available at *EURPUB* online.

## Supplementary Material

ckaa194_Supplementary_Data

## References

[1] Pongiglione B , De Stavola BL, Ploubidis GB. A systematic literature review of studies analyzing inequalities in health expectancy among the older population. PLoS One 2015;10:e0130747. 26115099 10.1371/journal.pone.0130747 PMC4482630

[2] Maki N , Martikainen P, Eikemo T, et al Educational differences in disability-free life expectancy: a comparative study of long-standing activity limitation in eight European countries. Soc Sci Med 2013;94:1–8. 23931939 10.1016/j.socscimed.2013.06.009

[3] Van Oyen H , Charafeddine R, Deboosere P, et al Contribution of mortality and disability to the secular trend in health inequality at the turn of century in Belgium. Eur J Public Health 2011;21:781–7. 21217118 10.1093/eurpub/ckq198

[4] Fries JF. Aging, natural death, and the compression of morbidity. N Engl J Med 1980;303:130–5. 7383070 10.1056/NEJM198007173030304

[5] Perez-Hernandez B , Rubio-Valverde JR, Nusselder WJ, Mackenbach JP. Socioeconomic inequalities in disability in Europe: contribution of behavioral, work-related and living conditions. Eur J Public Health 2019;29:640–7. 30753498 10.1093/eurpub/ckz009

[6] Mackenbach JP , Valverde JR, Bopp M, et al Determinants of inequalities in life expectancy: an international comparative study of eight risk factors. Lancet Public Health 2019;4:e529–e537. 31578987 10.1016/S2468-2667(19)30147-1

[7] Nusselder WJ , Looman CWN. Decomposition of differences in health expectancy by cause. Demography 2004;41:315–34. 15209043 10.1353/dem.2004.0017

[8] Reuser M , Bonneux LG, Willekens FJ. Smoking kills, obesity disables: a multistate approach of the US Health and Retirement Survey. Obesity (Silver Spring) 2009;17:783–9. 19165165 10.1038/oby.2008.640

[9] Klijs B , Mackenbach JP, Kunst AE. Obesity, smoking, alcohol consumption and years lived with disability: a Sullivan life table approach. BMC Public Health 2011;11:378. 21605473 10.1186/1471-2458-11-378 PMC3128016

[10] Nusselder WJ , Looman CWN, Franco OH, et al The relation between non-occupational physical activity and years lived with and without disability. J Epidemiol Community Health 2008;62:823–8. 18701734 10.1136/jech.2007.067165

[11] Van Oyen H , Berger N, Nusselder W, et al The effect of smoking on the duration of life with and without disability, Belgium 1997-2011. BMC Public Health 2014;14:723. 25026981 10.1186/1471-2458-14-723 PMC4223416

[12] Galobardes B , Lynch JW, Smith GD. Is the association between childhood socioeconomic circumstances and cause-specific mortality established? Update of a systematic review. J Epidemiol Community Health 2008;62:387–90. 18413449 10.1136/jech.2007.065508

[13] Galobardes B , Smith GD, Lynch JW. Systematic review of the influence of childhood socioeconomic circumstances on risk for cardiovascular disease in adulthood. Ann Epidemiol 2006;16:91–104. 16257232 10.1016/j.annepidem.2005.06.053

[14] Aldabe B , Anderson R, Lyly-Yrjanainen M, et al Contribution of material, occupational, and psychosocial factors in the explanation of social inequalities in health in 28 countries in Europe. J Epidemiol Community Health 2011;65:1123–31. 20584725 10.1136/jech.2009.102517 PMC3678208

[15] Standfeld SA. Social support and social cohesion. In: Marmot M, Wilkinson RG, editors. Social Determinants of Health. Oxford: Oxford University Press, 2006: 148–71.

[16] Hiscock R , Bauld L, Amos A, et al Socioeconomic status and smoking: a review. Ann N Y Acad Sci 2012;1248:107–23. 22092035 10.1111/j.1749-6632.2011.06202.x

[17] Devaux M , Sassi F. Alcohol Consumption and Harmful Drinking: Trends and Social Disparities across OECD Countries. Paris: OECD, 2015.

[18] Roskam AJ , Kunst AE, Van Oyen H, et al Comparative appraisal of educational inequalities in overweight and obesity among adults in 19 European countries. Int J Epidemiol 2010;39:392–404. 19926664 10.1093/ije/dyp329

[19] Beenackers MA , Kamphuis CB, Giskes K, et al Socioeconomic inequalities in occupational, leisure-time, and transport related physical activity among European adults: a systematic review. Int J Behav Nutr Phys Act 2012;9:116. 22992350 10.1186/1479-5868-9-116 PMC3491027

[20] Irala-Estevez JD , Groth M, Johansson L, et al A systematic review of socio-economic differences in food habits in Europe: consumption of fruit and vegetables. Eur J Clin Nutr 2000;54:706–14. 11002383 10.1038/sj.ejcn.1601080

[21] Marmot MG. Understanding social inequalities in health. Perspect Biol Med 2003;46:S9–23. 14563071

[22] Breen R , Jonsson JO. Inequality of opportunity in comparative perspective: recent research on educational attainment and social mobility. Annu Rev Sociol 2005;31:223–43.

[23] Huijts T , Stornes P, Eikemo TA, et al The social and behavioural determinants of health in Europe: findings from the European Social Survey (2014) special module on the social determinants of health. Eur J Public Health 2017;27:55–62. 28355646 10.1093/eurpub/ckw231

[24] Berger N , Robine JM, Ojima T, et al Harmonising summary measures of population health using global survey instruments. J Epidemiol Community Health 2016;70:1039–44. 27165845 10.1136/jech-2015-206870 PMC5036208

[25] Berger N , Van Oyen H, Cambois E, et al Assessing the validity of the Global Activity Limitation Indicator in fourteen European countries. BMC Med Res Methodol 2015;15:1. 25555466 10.1186/1471-2288-15-1 PMC4298058

[26] Cox B , van Oyen H, Cambois E, et al The reliability of the Minimum European Health Module. Int J Public Health 2009;54:55–60. 19183846 10.1007/s00038-009-7104-y

[27] Jagger C , Gillies C, Cambois E, et al The Global Activity Limitation Index measured function and disability similarly across European countries. J Clin Epidemiol 2010;63:892–9. 20171842 10.1016/j.jclinepi.2009.11.002

[28] Van Oyen H , Bogaert P, Yokota RTC, Berger N. Measuring disability: a systematic review of the validity and reliability of the Global Activity Limitations Indicator (GALI). Arch Public Health 2018;76:25. 29881544 10.1186/s13690-018-0270-8 PMC5985596

[29] Van Oyen H , Van der Heyden J, Perenboom R, Jagger C. Monitoring population disability: evaluation of a new Global Activity Limitation Indicator (GALI). Soz Präventiv Med 2006;51:153–61. 10.1007/s00038-006-0035-y 17191540

[30] Cabrero-Garcia J , Julia-Sanchis R. The Global Activity Limitation Index mainly measured functional disability, whereas self-rated health measured physical morbidity. J Clin Epidemiol 2014;67:468–76. 24411314 10.1016/j.jclinepi.2013.10.005

[31] Cabrero GJ , Julia-Sanchis R, Richart MM. Association of the global activity limitation indicator with specific measures of disability in adults aged below 65. Eur J Public Health 2020. 10.1093/eurpub/ckaa066 32408346

[32] Daly MC , Duncan GJ, McDonough P, Williams DR. Optimal indicators of socioeconomic status for health research. Am J Public Health 2002;92:1151–7. 12084700 10.2105/ajph.92.7.1151 PMC1447206

[33] Hoffmann R , Eikemo TA, Kulhanova I, et al The potential impact of a social redistribution of specific risk factors on socioeconomic inequalities in mortality: illustration of a method based on population attributable fractions. J Epidemiol Community Health 2013;67:56–62. 22760220 10.1136/jech-2011-200886

[34] Sullivan DF. A single index of mortality and morbidity. HSMHA Health Rep 1971;86:347–54. 5554262 PMC1937122

[35] Verbrugge LM , Jette AM. The disablement process. Soc Sci Med 1994;38:1–14. 8146699 10.1016/0277-9536(94)90294-1

[36] Nusselder WJ , Cambois EM, Wapperom D, et al Women’s excess unhealthy life years: disentangling the unhealthy life years gap. Eur J Public Health 2019;29:914–9. 31280299 10.1093/eurpub/ckz114 PMC6761840

[37] Rod NH , Lange T, Andersen I, et al Additive interaction in survival analysis use of the additive hazards model. Epidemiology 2012;23:733–7. 22732385 10.1097/EDE.0b013e31825fa218

