# Supplementary material for: Determinants of inequalities in years with disability: an international-comparative study
Source: Eur J Public Health. 2020 Nov 22;31(3):527–33. doi: 10.1093/eurpub/ckaa194 (PMC8277222; doi:10.1093/eurpub/ckaa194)
Supplement: ckaa194_Supplementary_Data [file ckaa194_supplementary_data.docx]

# Supplementary material

**Appendix S1. Overview of data sources for mortality**

| **Country** | **Design** | **Inclusion** | **Years** | **Person-years** | **Deaths** |
| --- | --- | --- | --- | --- | --- |
| Finland | Longitudinal | 80% sample | 2010-14 | 11644985 | 93850 |
| Sweden | Longitudinal | All | 2005-08 | 18238883 | 139884 |
| Norway | Longitudinal | All | 2006-09 | 9102575 | 86107 |
| Denmark | Longitudinal | All | 2010-14 | 15048315 | 127766 |
| England/Wales | Longitudinal | 1% sample | 2011-13 | 845053 | 5972 |
| Netherlands | Longitudinal |  | 2011-13 | 3736225 | 15612 |
| Belgium | Longitudinal | All | 2006-11 | 27184162 | 247709 |
| Austria | Longitudinal | All | 2010-14 | 9287635 | 67825 |
| Switzerland | Longitudinal | All nationals | 2011-13 | 12678229 | 81670 |
| France | Longitudinal | 1% sample | 2004-07 | 971147 | 7570 |
| Spain | Longitudinal | All | 2007-11 | 103180086 | 783281 |
| Hungary | Cross-sec. | All | 2010-12 | 16507050 | 232120 |
| Poland | Cross-sec. | All | 2010-12 | 56740803 | 658169 |
| Lithuania | Longitudinal | All | 2011-14 | 6273784 | 89212 |
| Estonia | Longitudinal | All | 2011-15 | 2751490 | 31450 |

**Appendix S2. Overview of survey data**

Appendix S3 additional information on the survey data from the Survey data from European Social Survey (ESS) and EU-Statistics on Income and Living Conditions (EU-SILC).

**Survey data from European Social Survey (ESS) and EU-Statistics on Income and Living Conditions (EU-SILC)**

|  | **European Social Survey (ESS)** | | **EU-Statistics on Income and Living Conditions**  **(EU-SILC)** | | | | |
| --- | --- | --- | --- | --- | --- | --- | --- |
| **Country** | **Analyses sample sizes** | **Individual Response Rate** | **Analyses sample sizes** | **Overall personal interview response rate** | | | |
|  |  | **2014** |  | **2005*** | **2009*** | **2010** | **2014** |
| Finland | 1587 | 62.67 | 32869 | - | - | 82.3 | 80.1 |
| Sweden | 1300 | 50.1 | 17498 | na | 73.00 | - | - |
| Norway | 1058 | 53.94 | 14709 | na | 60.40 | - | - |
| Denmark | 1111 | 51.85 | 18175 | - | - | 51.71 | na |
| England/Wales | 1734 | 43.56 | 23784 | - | - | 70.22 | 61.3 |
| Netherlands | 1507 | 58.61 | 30577 | - | - | 80.7 | 82 |
| Belgium | 1261 | 57.03 | 17414 | - | - | 62.28 | 98.96 |
| Austria | 1408 | 51.58 | 17220 | - | - | 75.58 | 76.57 |
| Switzerland | 1135 | 52.7 | 11356 | - | - | 74.44 | na |
| France | 1467 | 50.94 | 26226 | 83.02 | 78.10 | - | - |
| Spain | 1456 | 67.85 | 41924 | - | - | 80.55 | 77.71 |
| Hungary | 1343 | 52.7 | 29975 | - | - | 87.53 | 75.59 |
| Poland | 1186 | 65.84 | 42448 | - | - | 78.56 | 74.3 |
| Lithuania | 1765 | 68.87 | 16839 | - | - | 88.72 | 86.83 |
| Estonia | 1559 | 59.94 | 16772 | - | - | 79.76 | 70.81 |

Quality reports obtained from GESIS German Microdata Lab site:

https://www.gesis.org/en/missy/materials/EU-SILC/documents/quality-reports

The European Social Survey (ESS) is a biennial cross-national survey starting from 2001. It surveys beliefs, attitudes and behavior patterns of populations of more than 30 countries. The samples are representative of all individuals over 15 years old living in private households and have a minimum size of 1.500 individuals, except for countries with less than 2 million inhabitants.

The European Union Statistics on Living Conditions (EU-SILC) survey provides annual data on variables on poverty, income, social exclusion and living conditions. The survey was launched in 2003, and has extended its coverage to the 28 member states of the enlarged European Union. The target population is all private households and their members living in the country’s territory. All household members are surveyed and only those above 16 years are interviewed. EU-SILC provides both cross-sectional and longitudinal data, intermediate years are excluded to avoid including subject more than once. We included 28 countries from EU-SILC, with a total sample size of 603,785. For EU-SILC, we pooled years 2010 and 2014, except for Sweden, Norway and France, where we used 2005 and 2009 to better match with the mortality data. Because EU-SILC is a rotating panel survey, we did not include intermediate years to avoid including respondents multiple times*.*

**Appendix S3. Selection and classification of risk factors**

Appendix S3 gives additional information on the selection and classification of risk factors.

We used the following criteria for the selection of risk factors, in addition to whether the prevalence of the risk factor has been reported to differ between educational groups: (1) an estimate of the Relative Risk of mortality and disability is available in the literature, and (2) estimates of the prevalence of the risk factor by level of education in the 15 countries are available from internationally harmonized surveys. These criteria resulted in the selection of eight risk factors, and the exclusion of many other candidates. For example, we could not include housing and employment. Also, because the definition of the risk factor had to be identical for the Relative Risk estimates and the available prevalence data, exposure categories often had to be collapsed (e.g., dichotomized).

We selected the following risks factors:

- Father’s manual occupation as an indicator of the conditions in which adults with a lower and higher educational level have grown up [[1](#_ENREF_1)]
- low income as an indicator of current material living conditions [[2](#_ENREF_2)]
- few social contacts as an indicator of psychosocial conditions [[3](#_ENREF_3)]
- smoking [[4](#_ENREF_4)] as indicator of heath behaviours
- high alcohol consumption [[5](#_ENREF_5)] as indicator of heath behaviours
- high body-weight [[6](#_ENREF_6)] as indicator of heath behaviours
- low physical activity [[7](#_ENREF_7)] as indicator of heath behaviours
- low fruits and vegetables consumption [[8](#_ENREF_8)] as indicator of heath behaviours.

Together, these risk factors cover different but overlapping explanatory perspectives: whereas behavioural risk factors can be conceptualized as being ‘downstream’ in the causal pathway between level of education and mortality, father’s manual occupation and low income partly determine why lower and higher educated people have different health-related behaviours, and should therefore be seen as more ‘upstream’ [[9](#_ENREF_9)]. It is also important to note that father’s manual occupation partly determines a person’s educational achievement [[10](#_ENREF_10)], and should therefore, in contrast to the other risk factors, not be seen as a possible mediator of the effect of education on mortality, but as a factor capturing the persistent effect of childhood conditions on the risks of mortality and disability in later life.

Smoking was classified in three exposure categories: never (reference), former and current smokers. Income was classified in two exposure categories: lowest household income quintile versus income higher than lowest quintile (reference). Alcohol consumption was classified in three exposure categories: less than 25 gram alcohol per day (reference), between 25 and 45 gram alcohol per day, and more than 45 gram per day. Occupation of the father was classified in two exposure categories: manual and non-manual (reference). Fruit and vegetable was classified in two exposure categories: less than once a day fruit and vegetable consumption versus at least once a day fruit and vegetable consumption (reference). Body-weight was classification in three exposure categories: normal weight (BMI between 18.5 and <25 kg/m^2^ as reference), overweight (BMI between 25 and <30 kg/m^2^), obesity (BMI>=30 kg/m^2^). Physical activity was classified in two exposure categories: at least 5 days a week 30 minutes or longer of walking quickly, sports or other physical activity (reference) and versus less than 5 days a week 30 minutes or less of these activities.

**Appendix S4. Overview of Relative Risks for mortality and disability**

Appendix S4 gives the overview of the Relative Risks for mortality and disability and their sources.

**Overview of Relative Risks for mortality**

| Risk Factor | Level | Relative risk | Source* |
| --- | --- | --- | --- |
| Father's occupation | All other (=ref) | 1.0 | [[1](#_ENREF_1), [11-20](#_ENREF_11)] |
|  | Manual class | 1.1 |  |
| Income | All other (=ref) | 1.0 |  |
|  | Lowest quintile | 1.3 | [[21](#_ENREF_21)] |
| Social contact | All other (=ref) | 1.0 |  |
|  | less than once a week | 1.1 | [[22](#_ENREF_22)] |
| Smoking | Never (=ref) | 1.0 |  |
|  | Former | 1.3 | [[23](#_ENREF_23)] |
|  | Current | 2.2 | [[23](#_ENREF_23)] |
| Alcohol consumption | Average gr/day <24.9 | 1.0 |  |
|  | Average gr/day = 25-44.9 | 1.1 | [[24](#_ENREF_24)] |
|  | Average gr/day >=45 | 1.4 | [[24](#_ENREF_24)] |
| Overweight/obesity | Normal (BMI<25.0)(=ref) | 1.0 |  |
|  | Overweight (BMI=25.0-29.0) | 1.1 | [[25](#_ENREF_25)] |
|  | Obese (BMI>=30.0) | 1.7 | [[25](#_ENREF_25)] |
| Physical activity | At least 5 days per week (=ref) | 1.0 |  |
|  | Less than 5 days per week | 1.3 | [[23](#_ENREF_23)] |
| Fruit and vegetable consumption | At least once a day (=ref) | 1.0 |  |
|  | All other | 1.2 | [[26](#_ENREF_26)] |

*Where needed categories in the sources were pooled to reflect the exposure categories

|  |
| --- |

Relative risks for mortality were preferably based on meta-analyses or reviews. If these were not available we used individual studies. Where needed, we converted the relative risks for the exposure categories in the respective papers into the categories in our study that match with the exposure categories based on the ESS survey. The RR are adjusted for different sets of confounders, depending on the study. If more Relative Risks were presented in the literature, we selected Relative Risks adjusted for age and gender and other risk factors not on the causal pathway between the risk factor and mortality/disability, and for adult socioeconomic position. We averaged the selected relative risk for obtain the relative risk. We converted the RR into a beta by using ln(RR), averaged the beta’s, then converted the average beta into RR by using exp(beta). If the RR were separate for men and women, we first calculated average, following the same procedure.

**Overview of Relative Risks for disability**

| Risk Factor | Level | Relative risk | | Source* |
| --- | --- | --- | --- | --- |
| Father's occupation | All other (=ref) | 1 | |  |
|  | Manual class | 1.3 | | [[27-29](#_ENREF_27)] |
| Income | All other (=ref) | 1 | |  |
|  | Lowest quintile | 1.5 | | [[30-35](#_ENREF_30)] |
| Social contact | All other (=ref) | 1 | |  |
|  | Less than once a week | 1.05 | | Assumed same as for mortality |
| Smoking | Never (=ref) | 1 | |  |
|  | Former | 1.1 | |  |
|  | Current | 1.3 | |  |
| Alcohol consumption | Average gr/day <24.9) | 1 | |  |
|  | Average gr/day = 25-44.9) | 1 | | [[36-39](#_ENREF_36)] |
|  | Average gr/day >=45.0) | 1.05 | | [[36-39](#_ENREF_36)] |
| Body weight | Normal (BMI<25.0)(=ref) | 1 | |  |
|  | Overweight (BMI=25.0-29.0) | 1.4 | | [[38-42](#_ENREF_38)] |
|  | Obese (BMI>=30.0) | 1.8 | |  |
| Physical activity | At least 5 days per week (=ref) | 1 | |  |
|  | Less than 5 days per week | 1.5 | | [[43](#_ENREF_43)] |
| Fruit and vegetable consumption | At least once a day (=ref) | 1 | |  |
|  | All other | | 1.2 | [[36](#_ENREF_36)] |

*Where needed categories in the sources were pooled to reflect the exposure categories

Relative risks for disability were preferably based on reviews. If these were not available we used individual studies. Where needed, we converted the relative risks for the exposure categories in the respective papers into the categories in our study that match with the exposure categories based on the ESS survey. The RR are adjusted for different sets of confounders, depending on the study. If more Relative Risks were presented in the literature, we selected Relative Risks adjusted for age and gender and other risk factors not on the causal pathway between the risk factor and mortality/disability, and for adult socioeconomic position.

**Appendix S5. Educational differences and confidence intervals in years with disability**

Appendix S5 gives additional information about the 95%-confidence intervals to Table 1 in the main document.

Table 1. Years with disability^*^ and inequalities in years with disability (low-high) between ages35 and 80, by level of education by gender and country, around 2010

|  |  | |  | |  | **Men** | |  |  | |  |  | |
| --- | --- | --- | --- | --- | --- | --- | --- | --- | --- | --- | --- | --- | --- |
| **Country** | | **Low** | **95%-CI** | | **Mid** | **95%-CI** | | **High** | **95%-CI** | | **Gap** | **95%-CI** | |
| Finland | | 13.9 | 13.0 | 14.9 | 12.9 | 12.2 | 13.6 | 9.9 | 9.1 | 10.5 | 4.0 | 3.0 | 5.0 |
| Sweden | | 9.5 | 8.3 | 10.4 | 8.0 | 7.5 | 8.7 | 4.9 | 3.8 | 5.5 | 4.6 | 3.7 | 5.8 |
| Norway | | 10.4 | 9.3 | 11.6 | 7.9 | 7.1 | 8.5 | 3.8 | 3.2 | 4.5 | 6.6 | 5.3 | 7.7 |
| Denmark | | 11.4 | 9.9 | 12.4 | 10.7 | 9.8 | 11.4 | 9.4 | 8.4 | 10.4 | 2.0 | 1.0 | 3.6 |
| England & Wales | | 13.0 | 12.4 | 13.7 | 9.7 | 8.9 | 10.3 | 7.5 | 7.0 | 8.2 | 5.5 | 4.8 | 6.1 |
| Netherlands | | 13.4 | 12.3 | 14.5 | 11.2 | 10.5 | 12.0 | 8.9 | 8.0 | 9.7 | 4.5 | 3.3 | 5.5 |
| Belgium | | 13.6 | 12.7 | 14.3 | 8.9 | 8.2 | 9.5 | 6.2 | 5.4 | 6.6 | 7.3 | 6.6 | 8.2 |
| Austria | | 19.1 | 17.9 | 20.2 | 14.0 | 13.5 | 14.6 | 10.9 | 10.1 | 11.5 | 8.2 | 7.0 | 9.4 |
| Switzerland | | 13.4 | 11.6 | 15.3 | 10.2 | 9.5 | 10.9 | 8.1 | 7.4 | 8.8 | 5.3 | 3.4 | 7.2 |
| France | | 11.2 | 10.7 | 11.7 | 9.3 | 8.7 | 9.7 | 6.3 | 5.7 | 7.1 | 4.9 | 4.3 | 5.4 |
| Spain | | 11.2 | 10.9 | 11.4 | 9.0 | 8.5 | 9.7 | 7.0 | 6.1 | 7.2 | 4.1 | 3.9 | 4.4 |
| Hungary | | 13.8 | 13.1 | 14.4 | 11.9 | 11.5 | 12.2 | 8.6 | 7.9 | 9.2 | 5.2 | 4.5 | 5.8 |
| Poland | | 12.2 | 11.4 | 12.9 | 10.8 | 10.4 | 11.1 | 8.0 | 7.3 | 9.0 | 4.2 | 3.5 | 5.0 |
| Lithuania | | 11.9 | 10.3 | 13.6 | 9.1 | 8.6 | 9.6 | 6.9 | 6.1 | 7.8 | 5.0 | 3.4 | 6.7 |
| Estonia | | 16.2 | 15.3 | 17.5 | 13.9 | 13.3 | 14.5 | 12.6 | 11.8 | 13.5 | 3.7 | 2.4 | 4.6 |
| **Average** | | 12.3 | 12.1 | 12.6 | 9.9 | 9.7 | 10.1 | 7.4 | 7.1 | 7.6 | 4.9 | 4.7 | 5.2 |
|  | |  |  |  |  |  |  |  |  |  |  |  |  |
|  | | **Women** | | | | | | | | | | | |
| **Country** | | **Low** | **95%-CI** | | **Mid** | **95%-CI** | | **High** | **95%-CI** | | **Gap** | **95%-CI** | |
| Finland | | 16.2 | 14.9 | 17.3 | 16.4 | 15.8 | 17.5 | 13.7 | 13.1 | 14.4 | 2.5 | 1.4 | 3.8 |
| Sweden | | 14.0 | 12.5 | 15.9 | 11.3 | 10.5 | 11.9 | 8.8 | 8.0 | 10.0 | 5.1 | 3.2 | 6.6 |
| Norway | | 14.8 | 13.5 | 16.1 | 10.2 | 9.5 | 10.9 | 7.6 | 6.7 | 8.5 | 7.2 | 5.8 | 8.5 |
| Denmark | | 13.7 | 12.0 | 14.9 | 13.1 | 12.1 | 13.9 | 12.6 | 11.5 | 13.5 | 1.2 | 0.0 | 2.9 |
| England &Wales | | 14.4 | 13.7 | 15.0 | 10.5 | 9.9 | 11.1 | 9.2 | 8.7 | 10.0 | 5.2 | 4.5 | 5.8 |
| Netherlands | | 17.5 | 16.3 | 18.3 | 14.9 | 14.0 | 15.6 | 11.5 | 10.6 | 12.3 | 6.0 | 5.1 | 7.1 |
| Belgium | | 16.8 | 16.0 | 17.5 | 12.0 | 11.3 | 12.7 | 9.0 | 8.3 | 10.0 | 7.9 | 7.2 | 8.7 |
| Austria | | 19.3 | 18.3 | 20.1 | 14.7 | 14.1 | 15.2 | 12.5 | 11.4 | 13.4 | 6.8 | 6.0 | 7.7 |
| Switzerland | | 13.8 | 12.5 | 14.9 | 12.0 | 11.3 | 12.6 | 12.4 | 11.3 | 13.7 | 1.4 | 0.2 | 2.7 |
| France | | 13.6 | 13.1 | 14.0 | 10.8 | 10.1 | 11.2 | 8.5 | 7.2 | 9.1 | 5.1 | 4.8 | 5.7 |
| Spain | | 14.5 | 14.2 | 14.7 | 10.0 | 9.1 | 10.5 | 8.1 | 7.6 | 8.8 | 6.4 | 6.1 | 6.6 |
| Hungary | | 18.8 | 18.2 | 19.4 | 13.3 | 12.9 | 13.7 | 9.5 | 8.9 | 10.3 | 9.3 | 8.7 | 9.9 |
| Poland | | 15.2 | 14.7 | 16.2 | 12.8 | 12.4 | 13.1 | 10.2 | 9.6 | 11.0 | 5.1 | 4.1 | 5.6 |
| Lithuania | | 16.6 | 14.7 | 19.3 | 13.3 | 12.8 | 14.0 | 7.8 | 7.2 | 8.7 | 8.8 | 6.1 | 10.7 |
| Estonia | | 20.7 | 19.5 | 22.7 | 17.6 | 17.0 | 18.1 | 12.9 | 12.2 | 13.6 | 7.8 | 5.8 | 9.0 |
| **Average** | | 15.0 | 14.7 | 15.2 | 11.6 | 11.3 | 11.7 | 9.4 | 9.2 | 9.7 | 5.5 | 5.3 | 5.8 |

**Appendix S6. Educational inequalities in risk factor prevalence**

Appendix S1 gives Figure 1 in main document in colour and presents the same result as in table 1 in the main document, with added confidence intervals and information on the prevalence of the risk factor.

Figure 1 (in colour). Educational inequalities in risk factors expressed as prevalence ratio, by gender and country, ages 35-79 years

1. Men

1. Women

B Women

Note: Prevalence Ratio > 1 indicates a higher prevalence among the low educated. Average = population-weighted average of all European countries in the analysis.

Educational inequalities in risk factors expressed as prevalence ratio and age-standardized prevalence, by gender and country, ages 35-79 years

| **A. Men** |  |  |  |  |  |
| --- | --- | --- | --- | --- | --- |
| **Country** | **Risk Factor** | **Prevalence Ratio** | **95% CI** | **Age Standardised Prevalence** | **95% CI** |
| Finland | Father's manual occupation | 1.4 | [1.1,1.8] | 56.9 | [53.0,60.9] |
| Finland | Low income | 4.8 | [3.4,6.2] | 14.4 | [13.7,15.2] |
| Finland | Few social contacts | 1.6 | [1.1,2.2] | 39.9 | [36.3,43.4] |
| Finland | Smoking | 2.9 | [1.6,4.2] | 28.3 | [24.9,31.6] |
| Finland | High alcohol consumption | 0.8 | [0.1,1.5] | 10.3 | [8.1,12.6] |
| Finland | High bodyweight | 1.1 | [0.9,1.3] | 65.3 | [61.7,68.8] |
| Finland | Low physical activity | 1.1 | [0.9,1.4] | 66.1 | [62.7,69.6] |
| Finland | Low fruits and vegetables | 1.4 | [1.0,1.7] | 47.2 | [43.5,50.9] |
| Sweden | Father's manual occupation | 1.8 | [1.2,2.3] | 47.1 | [42.9,51.4] |
| Sweden | Low income | 3.1 | [2.1,4.2] | 12.3 | [11.3,13.3] |
| Sweden | Few social contacts | 1.3 | [0.8,1.7] | 29.3 | [25.7,32.8] |
| Sweden | Smoking | 4.0 | [0.8,7.2] | 15.3 | [12.3,18.2] |
| Sweden | High alcohol consumption | 1.1 | [0.5,1.7] | 14.0 | [11.2,16.7] |
| Sweden | High bodyweight | 1.5 | [1.2,1.8] | 66.1 | [62.3,69.8] |
| Sweden | Low physical activity | 1.1 | [1.0,1.3] | 68.3 | [64.6,72.0] |
| Sweden | Low fruits and vegetables | 1.6 | [1.3,2.0] | 58.9 | [55.0,62.7] |
| Norway | Father's manual occupation | 1.8 | [1.3,2.2] | 45.9 | [41.4,50.5] |
| Norway | Low income | 4.4 | [3.1,5.7] | 11.4 | [10.5,12.2] |
| Norway | Few social contacts | 1.2 | [0.8,1.5] | 35.0 | [30.8,39.2] |
| Norway | Smoking | 2.8 | [1.5,4.2] | 23.0 | [19.3,26.8] |
| Norway | High alcohol consumption | 0.8 | [0.4,1.3] | 13.2 | [10.2,16.2] |
| Norway | High bodyweight | 1.2 | [1.0,1.3] | 68.4 | [64.3,72.5] |
| Norway | Low physical activity | 1.0 | [0.9,1.2] | 71.9 | [68.0,75.9] |
| Norway | Low fruits and vegetables | 1.6 | [1.2,2.0] | 44.5 | [40.1,48.9] |
| Denmark | Father's manual occupation | 2.1 | [1.5,2.7] | 51.0 | [46.3,55.7] |
| Denmark | Low income | 3.4 | [1.5,5.2] | 13.3 | [12.3,14.3] |
| Denmark | Few social contacts | 1.0 | [0.7,1.3] | 33.4 | [29.1,37.6] |
| Denmark | Smoking | 1.6 | [1.0,2.2] | 26.4 | [22.3,30.4] |
| Denmark | High alcohol consumption | 1.3 | [0.8,1.9] | 21.7 | [17.9,25.5] |
| Denmark | High bodyweight | 1.4 | [1.2,1.7] | 59.5 | [55.0,63.9] |
| Denmark | Low physical activity | 0.9 | [0.8,1.0] | 65.5 | [61.2,69.8] |
| Denmark | Low fruits and vegetables | 1.7 | [1.3,2.1] | 55.0 | [50.5,59.4] |
| England/Wales | Father's manual occupation | 1.6 | [1.3,1.9] | 57.0 | [53.3,60.7] |
| England/Wales | Low income | 2.8 | [2.3,3.2] | 17.4 | [16.5,18.3] |
| England/Wales | Few social contacts | 1.0 | [0.8,1.2] | 47.7 | [44.2,51.2] |
| England/Wales | Smoking | 2.0 | [1.2,2.9] | 22.5 | [19.6,25.5] |
| England/Wales | High alcohol consumption | 1.1 | [0.8,1.5] | 30.8 | [27.6,34.1] |
| England/Wales | High bodyweight | 1.3 | [1.1,1.5] | 66.3 | [62.9,69.6] |
| England/Wales | Low physical activity | 1.2 | [1.0,1.4] | 60.8 | [57.3,64.2] |
| England/Wales | Low fruits and vegetables | 1.8 | [1.3,2.3] | 40.7 | [37.3,44.2] |
| Netherlands | Father's manual occupation | 1.7 | [1.2,2.1] | 46.3 | [42.3,50.4] |
| Netherlands | Low income | 2.7 | [1.6,3.8] | 16.1 | [15.3,17.0] |
| Netherlands | Few social contacts | 1.1 | [0.8,1.5] | 31.9 | [28.4,35.4] |
| Netherlands | Smoking | 2.0 | [1.2,2.8] | 31.2 | [27.7,34.8] |
| Netherlands | High alcohol consumption | 1.0 | [0.5,1.4] | 17.5 | [14.6,20.5] |
| Netherlands | High bodyweight | 1.3 | [1.1,1.6] | 59.9 | [56.1,63.6] |
| Netherlands | Low physical activity | 0.9 | [0.7,1.0] | 66.2 | [62.6,69.8] |
| Netherlands | Low fruits and vegetables | 1.2 | [0.9,1.5] | 45.8 | [41.9,49.6] |
| Belgium | Father's manual occupation | 2.2 | [1.6,2.8] | 53.4 | [49.0,57.9] |
| Belgium | Low income | 3.4 | [2.6,4.3] | 16.7 | [15.6,17.8] |
| Belgium | Few social contacts | 1.1 | [0.8,1.3] | 36.6 | [32.7,40.5] |
| Belgium | Smoking | 3.1 | [1.6,4.5] | 29.1 | [25.3,32.9] |
| Belgium | High alcohol consumption | 0.7 | [0.4,1.0] | 20.3 | [17.0,23.6] |
| Belgium | High bodyweight | 1.1 | [0.9,1.3] | 58.4 | [54.4,62.5] |
| Belgium | Low physical activity | 0.9 | [0.8,1.0] | 68.6 | [64.8,72.4] |
| Belgium | Low fruits and vegetables | 1.5 | [1.1,2.0] | 44.6 | [40.5,48.6] |
| Austria | Father's manual occupation | 2.1 | [1.4,2.8] | 51.9 | [47.7,56.0] |
| Austria | Low income | 2.6 | [2.0,3.2] | 17.1 | [15.9,18.3] |
| Austria | Few social contacts | 1.2 | [0.8,1.6] | 39.8 | [36.0,43.6] |
| Austria | Smoking | 1.8 | [1.1,2.6] | 33.8 | [30.2,37.5] |
| Austria | High alcohol consumption | 1.2 | [0.7,1.7] | 23.1 | [19.8,26.4] |
| Austria | High bodyweight | 1.4 | [1.1,1.7] | 65.5 | [61.8,69.2] |
| Austria | Low physical activity | 1.4 | [1.1,1.7] | 66.5 | [62.8,70.2] |
| Austria | Low fruits and vegetables | 1.0 | [0.8,1.1] | 62.3 | [58.6,66.0] |
| Switzerland | Father's manual occupation | 1.7 | [1.2,2.2] | 44.4 | [39.9,48.9] |
| Switzerland | Low income | 4.5 | [3.4,5.6] | 15.9 | [15.0,16.9] |
| Switzerland | Few social contacts | 1.1 | [0.8,1.4] | 38.2 | [34.1,42.3] |
| Switzerland | Smoking | 2.1 | [1.2,2.9] | 27.9 | [24.1,31.8] |
| Switzerland | High alcohol consumption | 0.9 | [0.5,1.3] | 16.9 | [13.7,20.1] |
| Switzerland | High bodyweight | 1.2 | [1.0,1.4] | 57.8 | [53.5,62.0] |
| Switzerland | Low physical activity | 0.8 | [0.7,0.9] | 63.1 | [59.0,67.3] |
| Switzerland | Low fruits and vegetables | 1.6 | [1.1,2.0] | 44.0 | [39.8,48.3] |
| France | Father's manual occupation | 2.3 | [1.5,3.2] | 53.1 | [49.0,57.3] |
| France | Low income | 4.7 | [3.6,5.8] | 16.9 | [16.0,17.8] |
| France | Few social contacts | 1.3 | [0.9,1.7] | 42.2 | [38.4,46.0] |
| France | Smoking | 2.2 | [1.2,3.1] | 31.4 | [27.9,34.9] |
| France | High alcohol consumption | 1.7 | [0.8,2.6] | 19.6 | [16.5,22.6] |
| France | High bodyweight | 1.4 | [1.0,1.7] | 60.7 | [56.9,64.4] |
| France | Low physical activity | 0.9 | [0.8,1.1] | 74.5 | [71.2,77.8] |
| France | Low fruits and vegetables | 1.3 | [0.9,1.6] | 46.5 | [42.9,50.2] |
| Spain | Father's manual occupation | 1.8 | [1.3,2.3] | 50.0 | [46.0,54.1] |
| Spain | Low income | 3.7 | [2.9,4.4] | 19.2 | [18.5,20.0] |
| Spain | Few social contacts | 1.1 | [0.8,1.5] | 27.0 | [23.6,30.4] |
| Spain | Smoking | 1.8 | [1.2,2.4] | 33.3 | [29.7,36.8] |
| Spain | High alcohol consumption | 1.7 | [0.8,2.5] | 19.5 | [16.5,22.6] |
| Spain | High bodyweight | 1.3 | [1.1,1.4] | 70.1 | [66.6,73.6] |
| Spain | Low physical activity | 0.9 | [0.8,1.1] | 61.5 | [57.8,65.3] |
| Spain | Low fruits and vegetables | 1.2 | [1.0,1.4] | 57.0 | [53.2,60.8] |
| Hungary | Father's manual occupation | 1.3 | [1.0,1.7] | 65.9 | [61.5,70.3] |
| Hungary | Low income | 16.0 | [8.9,23.1] | 19.0 | [18.0,19.9] |
| Hungary | Few social contacts | 1.1 | [0.9,1.3] | 72.0 | [68.2,75.8] |
| Hungary | Smoking | 2.8 | [1.5,4.2] | 44.9 | [40.5,49.2] |
| Hungary | High alcohol consumption | 1.2 | [0.6,1.8] | 21.1 | [17.5,24.8] |
| Hungary | High bodyweight | 0.9 | [0.8,1.1] | 71.9 | [68.0,75.8] |
| Hungary | Low physical activity | 1.0 | [0.9,1.2] | 81.2 | [77.8,84.6] |
| Hungary | Low fruits and vegetables | 1.2 | [1.0,1.5] | 76.3 | [72.6,80.0] |
| Poland | Father's manual occupation | 1.2 | [0.9,1.6] | 50.0 | [45.5,54.6] |
| Poland | Low income | 10.3 | [7.1,13.5] | 19.6 | [18.9,20.4] |
| Poland | Few social contacts | 0.9 | [0.7,1.0] | 66.2 | [62.1,70.2] |
| Poland | Smoking | 2.7 | [1.3,4.0] | 36.5 | [32.4,40.5] |
| Poland | High alcohol consumption | 1.5 | [0.4,2.6] | 13.6 | [10.7,16.5] |
| Poland | High bodyweight | 1.0 | [0.8,1.2] | 70.5 | [66.6,74.4] |
| Poland | Low physical activity | 0.9 | [0.7,1.0] | 61.4 | [57.2,65.5] |
| Poland | Low fruits and vegetables | 1.1 | [0.8,1.4] | 46.9 | [42.6,51.1] |
| Lithuania | Father's manual occupation | 1.2 | [0.9,1.6] | 59.9 | [55.5,64.3] |
| Lithuania | Low income | 9.0 | [4.5,13.5] | 17.7 | [16.4,19.0] |
| Lithuania | Few social contacts | 1.0 | [0.8,1.2] | 70.5 | [67.0,74.0] |
| Lithuania | Smoking | 1.6 | [1.1,2.2] | 49.9 | [46.0,53.8] |
| Lithuania | High alcohol consumption | 2.8 | [1.4,4.3] | 21.9 | [18.6,25.2] |
| Lithuania | High bodyweight | 1.1 | [0.9,1.3] | 68.3 | [64.6,71.9] |
| Lithuania | Low physical activity | 0.7 | [0.5,0.9] | 53.8 | [49.9,57.7] |
| Lithuania | Low fruits and vegetables | 1.9 | [1.4,2.4] | 61.5 | [57.7,65.2] |
| Estonia | Father's manual occupation | 1.3 | [1.0,1.7] | 59.8 | [55.6,64.1] |
| Estonia | Low income | 2.8 | [2.1,3.6] | 18.5 | [17.3,19.7] |
| Estonia | Few social contacts | 0.9 | [0.7,1.1] | 63.0 | [59.3,66.8] |
| Estonia | Smoking | 3.3 | [1.9,4.7] | 39.9 | [36.1,43.7] |
| Estonia | High alcohol consumption | 0.6 | [0.1,1.2] | 12.7 | [10.1,15.4] |
| Estonia | High bodyweight | 1.0 | [0.8,1.2] | 63.2 | [59.4,67.0] |
| Estonia | Low physical activity | 0.8 | [0.6,1.0] | 54.3 | [50.4,58.2] |
| Estonia | Low fruits and vegetables | 1.3 | [1.0,1.6] | 52.3 | [48.4,56.1] |
| Average | Father's manual occupation | 1.7 | [1.5,1.8] | 53.0 | [51.9,54.2] |
| Average | Low income | 4.0 | [3.7,4.4] | 16.8 | [16.6,17.1] |
| Average | Few social contacts | 1.0 | [0.9,1.1] | 43.8 | [42.8,44.9] |
| Average | Smoking | 2.3 | [1.9,2.7] | 30.3 | [29.4,31.3] |
| Average | High alcohol consumption | 1.2 | [1.0,1.4] | 20.8 | [19.9,21.6] |
| Average | High bodyweight | 1.2 | [1.1,1.3] | 65.4 | [64.4,66.4] |
| Average | Low physical activity | 1.0 | [1.0,1.1] | 65.7 | [64.7,66.7] |
| Average | Low fruits and vegetables | 1.4 | [1.3,1.6] | 49.0 | [47.9,50.0] |
|  |  |  |  |  |  |

|  |  |  |  |  |  |
| --- | --- | --- | --- | --- | --- |
| **Women** |  |  |  |  |  |
| **Country** | **Risk Factor** | **Prevalence Ratio** | **95% CI** | **Age standardized valence** | **95% CI** |
| Finland | Father's manual occupation | 1.5 | [1.1,1.9] | 56.4 | [52.4,60.4] |
| Finland | Low income | 3.9 | [2.8,4.9] | 15.4 | [14.6,16.2] |
| Finland | Few social contacts | 1.1 | [0.7,1.5] | 35.6 | [32.1,39.2] |
| Finland | Smoking | 4.6 | [2.1,7.2] | 21.8 | [18.7,24.9] |
| Finland | High alcohol consumption | 0.7 | [0.1,1.4] | 2.7 | [1.5,3.8] |
| Finland | High bodyweight | 1.6 | [1.2,2.1] | 51.7 | [47.9,55.5] |
| Finland | Low physical activity | 0.9 | [0.7,1.1] | 58.5 | [54.9,62.2] |
| Finland | Low fruits and vegetables | 2.5 | [1.5,3.6] | 31.6 | [28.2,35.0] |
| Sweden | Father's manual occupation | 1.9 | [1.4,2.4] | 54.5 | [50.2,58.9] |
| Sweden | Low income | 3.0 | [2.2,3.9] | 15.7 | [14.6,16.8] |
| Sweden | Few social contacts | 1.1 | [0.7,1.5] | 26.0 | [22.4,29.6] |
| Sweden | Smoking | 3.0 | [1.2,4.7] | 15.3 | [12.3,18.3] |
| Sweden | High alcohol consumption | 0.6 | [0.0,1.2] | 3.9 | [2.3,5.4] |
| Sweden | High bodyweight | 1.4 | [1.1,1.8] | 49.5 | [45.3,53.6] |
| Sweden | Low physical activity | 1.0 | [0.8,1.1] | 68.2 | [64.4,72.0] |
| Sweden | Low fruits and vegetables | 1.9 | [1.3,2.5] | 36.4 | [32.4,40.3] |
| Norway | Father's manual occupation | 2.0 | [1.5,2.5] | 48.9 | [44.3,53.5] |
| Norway | Low income | 4.6 | [3.4,5.7] | 15.4 | [14.4,16.3] |
| Norway | Few social contacts | 1.5 | [1.0,1.9] | 36.6 | [32.4,40.8] |
| Norway | Smoking | 5.3 | [2.3,8.3] | 22.9 | [19.2,26.7] |
| Norway | High alcohol consumption | 0.6 | [0.0,1.1] | 3.7 | [2.0,5.3] |
| Norway | High bodyweight | 1.5 | [1.2,1.9] | 45.5 | [41.0,50.0] |
| Norway | Low physical activity | 1.0 | [0.9,1.2] | 74.1 | [70.3,77.9] |
| Norway | Low fruits and vegetables | 1.5 | [1.0,2.0] | 29.6 | [25.5,33.7] |
| Denmark | Father's manual occupation | 1.7 | [1.3,2.1] | 52.1 | [47.7,56.4] |
| Denmark | Low income | 3.4 | [1.7,5.1] | 12.7 | [11.7,13.7] |
| Denmark | Few social contacts | 1.4 | [1.0,1.8] | 34.5 | [30.5,38.5] |
| Denmark | Smoking | 2.2 | [1.3,3.1] | 22.6 | [19.2,26.0] |
| Denmark | High alcohol consumption | 1.0 | [0.3,1.7] | 7.9 | [5.5,10.2] |
| Denmark | High bodyweight | 1.4 | [1.0,1.8] | 43.1 | [38.9,47.3] |
| Denmark | Low physical activity | 1.1 | [0.9,1.2] | 66.0 | [62.0,70.0] |
| Denmark | Low fruits and vegetables | 2.0 | [1.3,2.7] | 30.0 | [26.2,33.9] |
| England/Wales | Father's manual occupation | 2.3 | [1.7,2.8] | 50.4 | [46.8,54.1] |
| England/Wales | Low income | 3.0 | [2.6,3.5] | 18.9 | [17.9,19.8] |
| England/Wales | Few social contacts | 0.9 | [0.7,1.1] | 40.6 | [37.3,43.8] |
| England/Wales | Smoking | 2.7 | [1.6,3.9] | 20.3 | [17.6,23.0] |
| England/Wales | High alcohol consumption | 0.8 | [0.5,1.1] | 15.0 | [12.6,17.4] |
| England/Wales | High bodyweight | 1.2 | [1.0,1.5] | 52.3 | [48.8,55.7] |
| England/Wales | Low physical activity | 1.0 | [0.9,1.1] | 65.7 | [62.5,68.9] |
| England/Wales | Low fruits and vegetables | 2.5 | [1.7,3.3] | 32.3 | [29.2,35.4] |
| Netherlands | Father's manual occupation | 2.1 | [1.6,2.6] | 50.4 | [46.5,54.4] |
| Netherlands | Low income | 3.1 | [2.2,4.1] | 18.1 | [17.2,19.0] |
| Netherlands | Few social contacts | 1.2 | [0.8,1.5] | 28.0 | [24.7,31.3] |
| Netherlands | Smoking | 2.8 | [1.6,4.0] | 22.3 | [19.3,25.4] |
| Netherlands | High alcohol consumption | 2.2 | [0.4,4.0] | 5.4 | [3.7,7.1] |
| Netherlands | High bodyweight | 1.5 | [1.2,1.8] | 48.8 | [45.1,52.5] |
| Netherlands | Low physical activity | 1.0 | [0.9,1.1] | 63.9 | [60.3,67.4] |
| Netherlands | Low fruits and vegetables | 1.6 | [1.2,2.1] | 36.0 | [32.5,39.5] |
| Belgium | Father's manual occupation | 2.1 | [1.6,2.7] | 54.6 | [50.4,58.8] |
| Belgium | Low income | 4.3 | [3.4,5.2] | 19.0 | [17.8,20.2] |
| Belgium | Few social contacts | 1.3 | [0.9,1.7] | 36.5 | [32.7,40.2] |
| Belgium | Smoking | 3.1 | [1.8,4.4] | 26.6 | [23.1,30.1] |
| Belgium | High alcohol consumption | 0.8 | [0.2,1.4] | 6.9 | [5.0,8.8] |
| Belgium | High bodyweight | 1.2 | [0.9,1.5] | 44.0 | [40.1,47.8] |
| Belgium | Low physical activity | 0.9 | [0.8,1.0] | 75.0 | [71.5,78.4] |
| Belgium | Low fruits and vegetables | 1.4 | [1.0,1.8] | 35.3 | [31.5,39.0] |
| Austria | Father's manual occupation | 3.0 | [1.7,4.2] | 52.9 | [48.9,57.0] |
| Austria | Low income | 3.5 | [2.7,4.3] | 20.6 | [19.3,21.9] |
| Austria | Few social contacts | 1.4 | [1.0,1.8] | 43.8 | [40.0,47.5] |
| Austria | Smoking | 2.6 | [1.2,4.0] | 29.4 | [25.9,32.9] |
| Austria | High alcohol consumption | 0.6 | [0.0,1.2] | 4.3 | [2.7,5.8] |
| Austria | High bodyweight | 1.8 | [1.1,2.4] | 42.1 | [38.4,45.8] |
| Austria | Low physical activity | 1.3 | [1.1,1.6] | 68.6 | [65.1,72.1] |
| Austria | Low fruits and vegetables | 1.6 | [1.1,2.0] | 47.2 | [43.4,51.0] |
| Switzerland | Father's manual occupation | 1.8 | [1.2,2.4] | 45.8 | [41.5,50.1] |
| Switzerland | Low income | 3.0 | [2.4,3.6] | 19.7 | [18.7,20.7] |
| Switzerland | Few social contacts | 1.6 | [0.9,2.4] | 30.6 | [26.8,34.3] |
| Switzerland | Smoking | 1.6 | [0.8,2.5] | 25.0 | [21.5,28.6] |
| Switzerland | High alcohol consumption | 0.5 | [0.1,0.9] | 4.5 | [2.8,6.1] |
| Switzerland | High bodyweight | 2.0 | [1.1,2.9] | 33.8 | [29.9,37.7] |
| Switzerland | Low physical activity | 1.1 | [0.8,1.3] | 56.8 | [52.8,60.9] |
| Switzerland | Low fruits and vegetables | 1.0 | [0.6,1.4] | 21.6 | [18.2,25.0] |
| France | Father's manual occupation | 1.8 | [1.2,2.4] | 51.7 | [47.7,55.8] |
| France | Low income | 6.3 | [5.0,7.5] | 18.7 | [17.8,19.6] |
| France | Few social contacts | 2.1 | [1.2,3.0] | 38.1 | [34.5,41.6] |
| France | Smoking | 1.9 | [1.0,2.8] | 28.0 | [24.8,31.1] |
| France | High alcohol consumption | 0.8 | [0.1,1.6] | 3.6 | [2.2,4.9] |
| France | High bodyweight | 2.2 | [1.4,3.1] | 44.8 | [41.1,48.5] |
| France | Low physical activity | 1.0 | [0.8,1.1] | 80.8 | [78.0,83.6] |
| France | Low fruits and vegetables | 1.6 | [1.0,2.2] | 32.8 | [29.4,36.2] |
| Spain | Father's manual occupation | 1.4 | [1.1,1.8] | 49.0 | [45.0,53.1] |
| Spain | Low income | 3.7 | [3.0,4.3] | 19.7 | [19.0,20.5] |
| Spain | Few social contacts | 1.4 | [1.0,1.9] | 36.2 | [32.5,39.9] |
| Spain | Smoking | 1.8 | [1.2,2.5] | 29.0 | [25.5,32.4] |
| Spain | High alcohol consumption | 0.5 | [0.0,1.0] | 2.6 | [1.4,3.8] |
| Spain | High bodyweight | 1.5 | [1.1,1.9] | 46.6 | [42.8,50.4] |
| Spain | Low physical activity | 1.0 | [0.9,1.2] | 64.0 | [60.4,67.6] |
| Spain | Low fruits and vegetables | 1.3 | [1.0,1.7] | 42.0 | [38.3,45.7] |
| Hungary | Father's manual occupation | 1.5 | [1.2,1.8] | 67.6 | [63.7,71.6] |
| Hungary | Low income | 13.6 | [8.8,18.3] | 19.2 | [18.2,20.1] |
| Hungary | Few social contacts | 1.0 | [0.8,1.1] | 77.7 | [74.4,81.1] |
| Hungary | Smoking | 2.6 | [1.3,3.8] | 28.7 | [25.0,32.3] |
| Hungary | High alcohol consumption | 0.2 | [0.1,0.4] | 1.3 | [0.4,2.3] |
| Hungary | High bodyweight | 1.2 | [1.0,1.5] | 58.4 | [54.4,62.4] |
| Hungary | Low physical activity | 1.0 | [0.9,1.1] | 82.3 | [79.2,85.4] |
| Hungary | Low fruits and vegetables | 1.4 | [1.1,1.6] | 71.8 | [68.2,75.4] |
| Poland | Father's manual occupation | 1.0 | [0.8,1.2] | 56.1 | [51.9,60.3] |
| Poland | Low income | 10.8 | [8.2,13.3] | 19.4 | [18.7,20.1] |
| Poland | Few social contacts | 1.0 | [0.9,1.2] | 65.6 | [61.8,69.4] |
| Poland | Smoking | 1.9 | [1.0,2.8] | 24.3 | [20.8,27.7] |
| Poland | High alcohol consumption | 1.0 | [0.7,2.7] | 1.3 | [0.4,2.2] |
| Poland | High bodyweight | 1.2 | [0.9,1.4] | 49.8 | [45.9,53.7] |
| Poland | Low physical activity | 1.1 | [0.9,1.3] | 64.5 | [60.7,68.3] |
| Poland | Low fruits and vegetables | 2.2 | [1.3,3.1] | 33.0 | [29.2,36.7] |
| Lithuania | Father's manual occupation | 1.1 | [0.8,1.4] | 56.4 | [52.4,60.4] |
| Lithuania | Low income | 6.3 | [4.2,8.3] | 19.5 | [18.3,20.8] |
| Lithuania | Few social contacts | 1.1 | [0.9,1.2] | 71.7 | [68.6,74.8] |
| Lithuania | Smoking | 2.2 | [0.8,3.6] | 17.6 | [15.0,20.3] |
| Lithuania | High alcohol consumption | 1.6 | [0.3,3.5] | 3.3 | [2.0,4.7] |
| Lithuania | High bodyweight | 1.2 | [0.9,1.4] | 57.4 | [54.0,60.7] |
| Lithuania | Low physical activity | 0.8 | [0.7,1.0] | 63.0 | [59.8,66.3] |
| Lithuania | Low fruits and vegetables | 2.4 | [1.7,3.0] | 47.2 | [43.9,50.6] |
| Estonia | Father's manual occupation | 0.9 | [0.7,1.1] | 59.9 | [56.0,63.9] |
| Estonia | Low income | 2.4 | [1.9,2.9] | 19.5 | [18.4,20.6] |
| Estonia | Few social contacts | 1.1 | [0.9,1.3] | 62.5 | [59.0,65.9] |
| Estonia | Smoking | 3.6 | [2.1,5.2] | 22.1 | [19.1,25.1] |
| Estonia | High alcohol consumption | 4.7 | [2.5,12.0] | 1.7 | [0.8,2.7] |
| Estonia | High bodyweight | 1.3 | [1.1,1.6] | 52.4 | [49.0,55.7] |
| Estonia | Low physical activity2 | 1.1 | [0.9,1.3] | 54.7 | [51.1,58.2] |
| Estonia | Low fruits and vegetables | 1.6 | [1.2,2.1] | 35.5 | [32.2,38.9] |
| Average | Father's manual occupation | 1.6 | [1.5,1.8] | 52.5 | [51.4,53.6] |
| Average | Low income | 4.1 | [3.9,4.4] | 18.2 | [18.0,18.5] |
| Average | Few social contacts | 1.1 | [1.0,1.2] | 43.0 | [42.0,44.0] |
| Average | Smoking | 2.4 | [2.0,2.8] | 24.9 | [24.0,25.8] |
| Average | High alcohol consumption | 0.6 | [0.4,0.7] | 5.9 | [5.4,6.4] |
| Average | High bodyweight | 1.4 | [1.2,1.5] | 47.9 | [46.9,48.9] |
| Average | Low physical activity | 1.0 | [1.0,1.1] | 69.1 | [68.2,70.1] |
| Average | Low fruits and vegetables | 1.8 | [1.6,2.1] | 35.8 | [34.8,36.8] |

**Appendix S7. Effect of ‘upward leveling’ on years with disability**

Appendix S7 gives Figure 2 in main document in colour and presents the same result in a table with added confidence intervals.

Figure 2 (in collor) Effect of ‘upward levelling’ of risk factor prevalence on years with disability between age 35 and 80 years, by gender and country

**
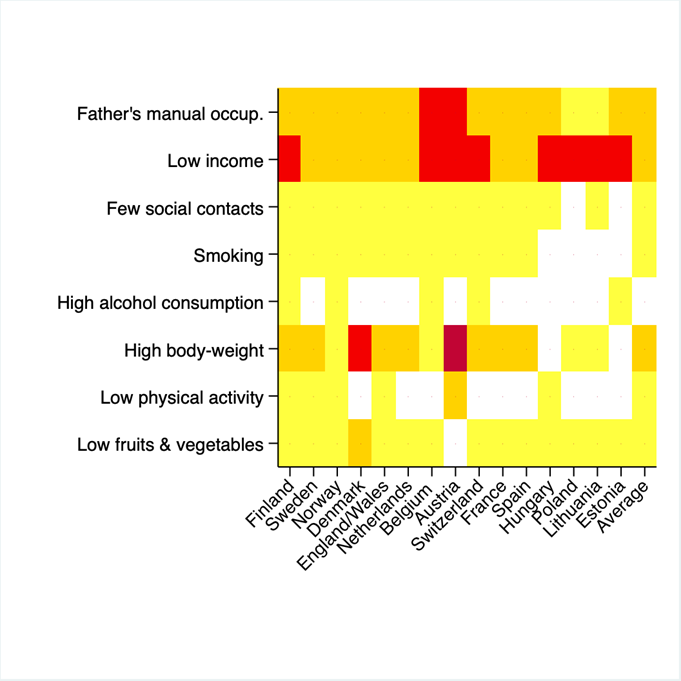
**


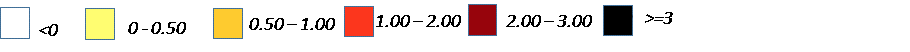


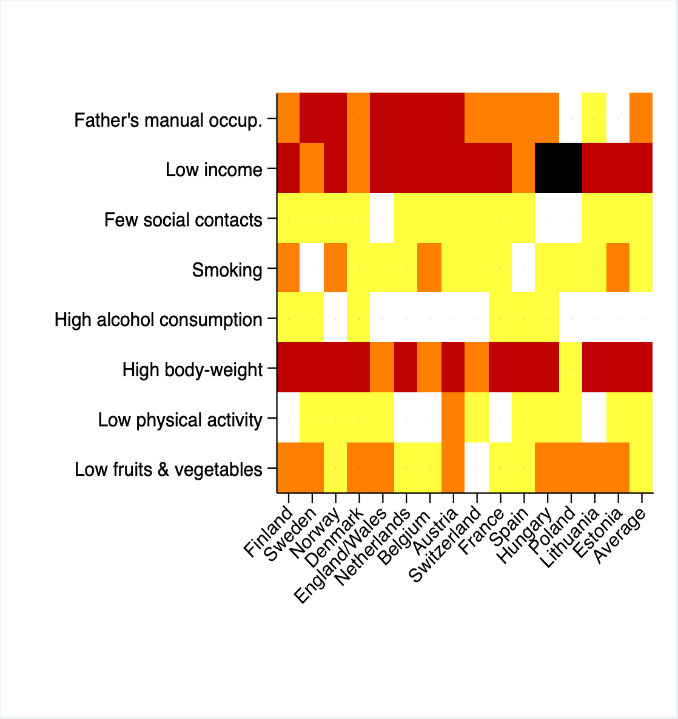


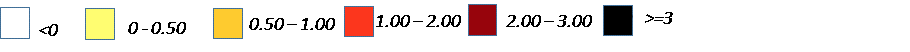


Table S7. Effect of ‘upward levelling’ of risk factor prevalence on years with disability between age 35 and 80 years, by gender and country

| Gender | Country | Father  Occupation | Income | Low social contact | Physical Activity | Fruit &Vegetable | Smoking | high body weight | High alcohol consumption |
| --- | --- | --- | --- | --- | --- | --- | --- | --- | --- |
| Men | *Finland* | *0.58* | *1.24* | *0.10* | *0.22* | *0.26* | *0.24* | *0.63* | *0.02* |
|  |  | *[0.53,0.63]* | *[1.04,1.45]* | *[0.05,0.17]* | *[-0.09,0.58]* | *[-0.02,0.53]* | *[0.06,0.41]* | *[0.15,1.47]* | *[0.02,0.02]* |
|  | *Sweden* | *0.55* | *0.55* | *0.02* | *0.13* | *0.35* | *0.25* | *0.88* | *0.00* |
|  |  | *[0.46,0.61]* | *[0.34,0.68]* | *[-0.01,0.06]* | *[-0.07 0.41]* | *[0.19,0.50]* | *[0.10,0.34]* | *[0.48,1.26]* | *[0.00,0.00]* |
|  | *Norway* | *0.61* | *0.62* | *0.01* | *0.07* | *0.37* | *0.34* | *0.31* | *0.01* |
|  |  | *[0.53,0.70]* | *[0.46,0.79]* | *[-0.01,0.05]* | *[-0.17,0.20]* | *[0.13,0.43]* | *[0.19,0.41]* | *[0.08,0.76]* | *[0.00,0.00]* |
|  | *Denmark* | *0.88* | *0.73* | *0.01* | *-0.17* | *0.57* | *0.33* | *1.03* | *-0.01* |
|  |  | *[0.87,0.88]* | *[0.72,0.73]* | *[0.01,0.01]* | *[-0.17,-0.17]* | *[0.56,0.57]* | *[0.32,0.34]* | *[1.03,1.04]* | *[-0.04,0.00]* |
|  | *UK* | *0.76* | *0.91* | *0.01* | *0.27* | *0.46* | *0.30* | *0.72* | *0.00* |
|  |  | *[0.71,0.81]* | *[0.79,1.06]* | *[-0.05,0.03]* | *[-0.13,0.47]* | *[0.17,0.58]* | *[0.14,0.43]* | *[0.20,1.15]* | *[0.00,0.00]* |
|  | *Netherlands* | *0.72* | *0.96* | *0.02* | *-0.11* | *0.16* | *0.38* | *0.86* | *0.00* |
|  |  | *[0.65,0.80]* | *[0.74,1.24]* | *[-0.03,0.07]* | *[-0.48,0.02]* | *[-0.27,0.21]* | *[0.17,0.51]* | *[0.19,1.26]* | *[0.00,0.00]* |
|  | *Belgium* | *1.08* | *1.15* | *0.01* | *-0.16* | *0.33* | *0.33* | *0.40* | *0.02* |
|  |  | *[1.00,1.15]* | *[0.96,1.31]* | *[-0.03,0.06]* | *[-0.51,0.03]* | *[0.14,0.52]* | *[0.13,0.42]* | *[-0.04,1.04]* | *[0.00,0.02]* |
|  | *Austria* | *1.41* | *1.52* | *0.05* | *0.97* | *-0.06* | *0.28* | *2.18* | *-0.01* |
|  |  | *[1.30,1.51]* | *[1.28,1.89]* | *[-0.02,0.12]* | *[0.26,1.04]* | *[-0.36,0.21]* | *[0.09,0.54]* | *[0.88,2.30]* | *[-0.01,-0.01]* |
|  | *Switzerland* | *0.70* | *1.32* | *0.06* | *-0.24* | *0.23* | *0.32* | *0.84* | *0.01* |
|  |  | *[0.57,0.82]* | *[0.90,1.68]* | *[-0.04,0.07]* | *[-0.73,-0.14]* | *[0.18,0.67]* | *[0.05,0.39]* | *[0.06,1.27]* | *[0.00,0.00]* |
|  | *France* | *0.91* | *0.87* | *0.03* | *0.00* | *0.16* | *0.09* | *0.75* | *-0.03* |
|  |  | *[0.86,0.96]* | *[0.76,0.97]* | *[-0.02,0.06]* | *[-0.38,0.06]* | *[0.04,0.32]* | *[-0.01,0.25]* | *[0.30,1.20]* | *[-0.03,-0.03]* |
|  | *Spain* | *0.64* | *0.76* | *0.01* | *-0.05* | *0.17* | *0.16* | *0.65* | *-0.01* |
|  |  | *[0.62,0.66]* | *[0.71,0.84]* | *[-0.01,0.07]* | *[-0.29,0.12]* | *[0.00,0.37]* | *[0.01,0.27]* | *[0.31,1.03]* | *[-0.01,-0.01]* |
|  | *Hungary* | *0.51* | *1.54* | *0.01* | *0.09* | *0.25* | *-0.09* | *-0.40* | *-0.01* |
|  |  | *[0.48,0.54]* | *[1.34,1.68]* | *[-0.01,0.04]* | *[-0.14,0.19]* | *[0.04,0.40]* | *[-0.31,0.03]* | *[-0.73,-0,10]* | *[-0.03,-0.03]* |
|  | *Poland* | *0.24* | *1.42* | *-0.03* | *-0.11* | *0.06* | *-0.08* | *0.25* | *-0.03* |
|  |  | *[0.21,0.26]* | *[1.23,1.59]* | *[-0.07,0.00]* | *[-0.39,0.02]* | *[-0.11,0.27]* | *[-0.22,0.13]* | *[-0.14,0.69]* | *[-0.03,-0.03]* |
|  | *Lithuania* | *0.34* | *1.27* | *0.01* | *-0.19* | *0.50* | *-0.03* | *0.30* | *-0.18* |
|  |  | *[0.28,0.40]* | *[0.90,1.67]* | *[-0.01,0.03]* | *[-0.58,-0.08]* | *[0.26,0.64]* | *[-0.19,0.12]* | *[0.21,1.14]* | *[-0.21,-0.16]* |
|  | *Estonia* | *0.59* | *1.24* | *-0.03* | *-0.29* | *0.30* | *-0.07* | *-0.04* | *0.03* |
|  |  | *[0.55,0.66]* | *[0.98,1.54]* | *[-0.05,0.03]* | *[-0.60,-0.06]* | *[0.04,0.51]* | *[-0.32,0.15]* | *[-0.50,0.71]* | *[0.03,0.03]* |
|  | *Average* | *0.70* | *1.00* | *0.01* | *0.05* | *0.25* | *0.17* | *0.64* | *-0.01* |
|  | *Average* | *[0.68,0.71]* | *[0.96,1.05]* | *[-0.01,0.03]* | *[-0.01,0.11]* | *[0.17,0.32]* | *[0.10,0.21]* | *[0.44,0.81]* | *[-0.02,-0.01]* |

| Gender | Country | Father  Occupation | Income | Low social contact | Physical Activity | Fruit &Vegetable | Smoking | high body weight | High alcohol consumption |
| --- | --- | --- | --- | --- | --- | --- | --- | --- | --- |
| Women | *Finland* | *0.81* | *1.41* | *0.02* | *-0.14* | *0.72* | *0.59* | *1.47* | *0.00* |
|  |  | *[0.72,0.88]* | *[1.03,1.64]* | *[-0.08,0.10]* | *[-0.85,0.31]* | *[0.30,1.07]* | *[0.13,1.07]* | *[0.23,2.31]* | *[0.00,0.00]* |
|  | *Sweden* | *1.05* | *0.93* | *0.00* | *0.11* | *0.68* | *-0.21* | *1.35* | *0.00* |
|  |  | *[0.91,1.22]* | *[0.53,1.18]* | *[-0.07,0.06]* | *[-0.34,0.33]* | *[0.27,0.76]* | *[0.03,0.48]* | *[0.40,1.72]* | *[0.00,0.00]* |
|  | *Norway* | *1.18* | *1.37* | *0.12* | *0.23* | *0.45* | *0.78* | *1.32* | *0.00* |
|  |  | *[1.06,1.31]* | *[1.31,1.86]* | *[0.04,0.18]* | *[-0.17,0.43]* | *[0.08,0.50]* | *[0.52,0.94]* | *[0.68,1.93]* | *[0.00,0.00]* |
|  | *Denmark* | *0.82* | *0.91* | *0.07* | *0.17* | *0.61* | *0.35* | *1.20* | *0.00* |
|  |  | *[0.70,0.90]* | *[0.64,1.24]* | *[0.01,0.11]* | *[-0.10,0.43]* | *[0.14,0.54]* | *[0.10,0.50]* | *[0.40,1.36]* | *[0.00,0.00]* |
|  | *UK* | *1.30* | *1.21* | *-0.01* | *0.07* | *0.56* | *0.45* | *0.75* | *0.00* |
|  |  | *[1.23,1.38]* | *[1.07,1.38]* | *[-0.06,0.03]* | *[0.11,0.90]* | *[0.40,0.74]* | *[0.35,0.75]* | *[0.09,1.22]* | *[0.00,0.00]* |
|  | *Netherlands* | *1.39* | *1.53* | *0.05* | *-0.01* | *0.45* | *0.45* | *1.90* | *0.00* |
|  |  | *[1.28,1.48]* | *[1.24,1.84]* | *[-0.02,0.11]* | *[-0.65,0.16]* | *[0.20,0.68]* | *[0.27,0.82]* | *[1.00,2.43]* | *[0.00,0.00]* |
|  | *Belgium* | *1.48* | *1.86* | *0.07* | *-0.17* | *0.30* | *0.68* | *0.95* | *0.00* |
|  |  | *[1.39,1.55]* | *[1.67,2.11]* | *[0.02,0.18]* | *[-0.41,0.31]* | *[0.07,0.56]* | *[0.41,0.90]* | *[-0.07,1.58]* | *[0.00,0.00]* |
|  | *Austria* | *1.98* | *1.86* | *0.12* | *0.67* | *0.67* | *0.46* | *1.81* | *0.00* |
|  |  | *[1.87,2.08]* | *[1.61,2.16]* | *[0.02,0.20]* | *[0.15,1.19]* | *[0.24,1.00]* | *[0.27,0.86]* | *[0.92,2.60]* | *[0.00,0.00]* |
|  | *Switzerland* | *0.86* | *1.35* | *0.08* | *0.15* | *-0.03* | *0.37* | *0.91* | *0.00* |
|  |  | *[0.76,0.95]* | *[1.12,1.77]* | *[0.01,0.13]* | *[-0.20,0.67]* | *[-0.25,0.63]* | *[0.11,1.06]* | *[-0.47,1.59]* | *[0.00,0.00]* |
|  | *France* | *0.82* | *1.41* | *0.12* | *-0.15* | *0.27* | *0.05* | *1.90* | *0.00* |
|  |  | *[0.78,0.84]* | *[1.20,1.45]* | *[0.07,0.19]* | *[-0.72,0.10]* | *[0.06,0.56]* | *[-0.72,-0.08]* | *[1.15,2.33]* | *[0.00,0.00]* |
|  | *Spain* | *0.53* | *1.00* | *0.06* | *0.20* | *0.31* | *-0.10* | *1.35* | *0.00* |
|  |  | *[0.52,0.55]* | *[0.92,1.09]* | *[0.01,0.18]* | *[-0.75,0.42]* | *[-0.18,0.41]* | *[-0.37,0.27]* | *[0.66,2.24]* | *[0.00,0.00]* |
|  | *Hungary* | *1.00* | *2.33* | *0.00* | *0.07* | *0.68* | *0.42* | *1.04* | *0.01* |
|  |  | *[0.96,1.04]* | *[2.18,2.57]* | *[-0.10,0.02]* | *[-0.16,0.38]* | *[0.23,0.81]* | *[0.24,0.56]* | *[0.01,1.58]* | *[0.00,0.01]* |
|  | *Poland* | *-0.08* | *2.05* | *-0.01* | *0.36* | *0.59* | *0.15* | *0.36* | *0.00* |
|  | *Poland* | *[-0.11,-0.05]* | *[1.89,2.31]* | *[-0.11,0.11]* | *[-0.61,0.67]* | *[0.33,1.32]* | *[-0.17,0.45]* | *[-1.23,2.70]* | *[0.00,0.00]* |
|  | *Lithuania* | *0.20* | *1.69* | *0.03* | *-0.26* | *0.91* | *0.10* | *1.27* | *-0.01* |
|  |  | *[0.17,0.24]* | *[1.20,2.16]* | *[-0.02,0.09]* | *[-0.65,0.02]* | *[0.55,1.20]* | *[-0.08,0.18]* | *[0.48,2.58]* | *[-0.01,-0.01]* |
|  | *Estonia* | *-0.27* | *1.73* | *0.06* | *0.18* | *0.75* | *0.67* | *1.68* | *-0.01* |
|  |  | *[-0.31,-0.25]* | *[1.32,2.18]* | *[-0.03,0.12]* | *[-0.38,0.60]* | *[0.21,0.93]* | *[0.21,0.95]* | *[0.96,3.09]* | *[-0.02,-0.01]* |
|  | *Average* | *0.86* | *1.42* | *0.04* | *0.10* | *0.45* | *0.23* | *1.19* | *0.00* |
|  |  | *[0.84,0.88]* | *[1.36,1.49]* | *[0.02,0.07]* | *[-0.11,0.22]* | *[0.31,0.55]* | *[0.10,0.28]* | *[0.83,1.48]* | *[0.00,0.00]* |

**References in appendices**

1 Galobardes B, Lynch JW, Smith GD. Is the association between childhood socioeconomic circumstances and cause-specific mortality established? Update of a systematic review. *J Epidemiol Community Health* 2008;**62**:387-90.

2 Aldabe B, Anderson R, Lyly-Yrjanainen M*, et al.* Contribution of material, occupational, and psychosocial factors in the explanation of social inequalities in health in 28 countries in Europe. *J Epidemiol Community Health* 2011;**65**:1123-31.

3 Standfeld SA. Social support and social cohesion. In: Marmot M, Wilkinson RG, eds. *Social Determinants of health*. Oxford: Osford University Press 2006:148-71.

4 Hiscock R, Bauld L, Amos A*, et al.* Socioeconomic status and smoking: a review. *Ann N Y Acad Sci* 2012;**1248**:107-23.

5 Devaux M, Sassi F. Alcohol consumption and harmful drinking: trends and social disparities across OECD countries. Paris: OECD 2015.

6 Roskam AJ, Kunst AE, Van Oyen H*, et al.* Comparative appraisal of educational inequalities in overweight and obesity among adults in 19 European countries. *Int J Epidemiol* 2010;**39**:392-404.

7 Beenackers MA, Kamphuis CB, Giskes K*, et al.* Socioeconomic inequalities in occupational, leisure-time, and transport related physical activity among European adults: a systematic review. *Int J Behav Nutr Phys Act* 2012;**9**:116.

8 Irala-Estevez JD, Groth M, Johansson L*, et al.* A systematic review of socio-economic differences in food habits in Europe: consumption of fruit and vegetables. *Eur J Clin Nutr* 2000;**54**:706-14.

9 Marmot MG. Understanding social inequalities in health. *Perspect Biol Med* 2003;**46**:S9-23.

10 Breen R, Jonsson JO. Inequality of opportunity in comparative perspective: Recent research on educational attainment and social mobility. *Annu Rev Sociol* 2005;**31**:223-43.

11 Elo IT, Martikainen P, Myrskyla M. Socioeconomic status across the life course and all-cause and cause-specific mortality in Finland. *Soc Sci Med* 2014;**119**:198-206.

12 Hayward MD, Gorman BK. The long arm of childhood: the influence of early-life social conditions on men's mortality. *Demography* 2004;**41**:87-107.

13 Juarez SP, Goodman A, Koupil I. From cradle to grave: tracking socioeconomic inequalities in mortality in a cohort of 11 868 men and women born in Uppsala, Sweden, 1915-1929. *J Epidemiol Commun H* 2016;**70**:569-75.

14 Kelly-Irving M, Lepage B, Dedieu D*, et al.* Adverse childhood experiences and premature all-cause mortality. *Eur J Epidemiol* 2013;**28**:721-34.

15 Khang YH. Relationship between childhood socio-economic position and mortality risk in adult males of the Korea Labour and Income Panel Study (KLIPS). *Public Health* 2006;**120**:724-31.

16 Lawlor DA, Sterne JA, Tynelius P*, et al.* Association of childhood socioeconomic position with cause-specific mortality in a prospective record linkage study of 1,839,384 individuals. *Am J Epidemiol* 2006;**164**:907-15.

17 Power C, Hypponen E, Smith GD. Socioeconomic position in childhood and early adult life and risk of mortality: a prospective study of the mothers of the 1958 British birth cohort. *Am J Public Health* 2005;**95**:1396-402.

18 Strand BH, Kunst A. Childhood socioeconomic position and cause-specific mortality in early adulthood. *Am J Epidemiol* 2007;**165**:85-93.

19 Stringhini S, Dugravot A, Kivimaki M*, et al.* Do different measures of early life socioeconomic circumstances predict adult mortality? Evidence from the British Whitehall II and French GAZEL studies. *J Epidemiol Commun H* 2011;**65**:1097-103.

20 Galobardes B, Lynch JW, Davey Smith G. Childhood socioeconomic circumstances and cause-specific mortality in adulthood: systematic review and interpretation. *Epidemiol Rev* 2004;**26**:7-21.

21 Martikainen P, Makela P, Koskinen S*, et al.* Income differences in mortality: a register-based follow-up study of three million men and women. *Int J Epidemiol* 2001;**30**:1397-405.

22 Shor E, Roelfs DJ. Social contact frequency and all-cause mortality: a meta-analysis and meta-regression. *Soc Sci Med* 2015;**128**:76-86.

23 Stringhini S, Carmeli C, Jokela M*, et al.* Socioeconomic status and the 25 x 25 risk factors as determinants of premature mortality: a multicohort study and meta-analysis of 1.7 million men and women. *Lancet* 2017;**389**:1229-37.

24 Stockwell T, Zhao J, Panwar S*, et al.* Do "Moderate" Drinkers Have Reduced Mortality Risk? A Systematic Review and Meta-Analysis of Alcohol Consumption and All-Cause Mortality. *J Stud Alcohol Drugs* 2016;**77**:185-98.

25 Di Angelantonio E, Bhupathiraju SN, Wormser D*, et al.* Body-mass index and all-cause mortality: individual-participant-data meta-analysis of 239 prospective studies in four continents. *Lancet* 2016;**388**:776-86.

26 Wang X, Ouyang Y, Liu J*, et al.* Fruit and vegetable consumption and mortality from all causes, cardiovascular disease, and cancer: systematic review and dose-response meta-analysis of prospective cohort studies. *BMJ* 2014;**349**:g4490.

27 Guralnik JM, Butterworth S, Wadsworth ME*, et al.* Childhood socioeconomic status predicts physical functioning a half century later. *J Gerontol A Biol Sci Med Sci* 2006;**61**:694-701.

28 Montez JK, Hayward MD. Cumulative childhood adversity, educational attainment, and active life expectancy among U.S. adults. *Demography* 2014;**51**:413-35.

29 Osler M, Madsen M, Nybo Andersen AM*, et al.* Do childhood and adult socioeconomic circumstances influence health and physical function in middle-age? *Soc Sci Med* 2009;**68**:1425-31.

30 Broese van Groenou MI, Deeg DJ, Penninx BW. Income differentials in functional disability in old age: relative risks of onset, recovery, decline, attrition and mortality. *Aging Clin Exp Res* 2003;**15**:174-83.

31 Cambois E, Sole-Auro A, Robine JM. Economic Hardship and Educational Differentials in Disability in 26 European Countries. *J Aging Health* 2016;**28**:1214-38.

32 Fuller-Thomson E, Gadalla T. Income inequality and limitations in activities of daily living: a multilevel analysis of the 2003 American Community Survey. *Public Health* 2008;**122**:221-8.

33 Nordstrom CK, Diez Roux AV, Schulz R*, et al.* Socioeconomic position and incident mobility impairment in the Cardiovascular Health Study. *BMC Geriatr* 2007;**7**:11.

34 Rueda S, Artazcoz L, Navarro V. Health inequalities among the elderly in western Europe. *J Epidemiol Community Health* 2008;**62**:492-8.

35 von dem Knesebeck O, Luschen G, Cockerham WC*, et al.* Socioeconomic status and health among the aged in the United States and Germany: a comparative cross-sectional study. *Soc Sci Med* 2003;**57**:1643-52.

36 Artaud F, Dugravot A, Sabia S*, et al.* Unhealthy behaviours and disability in older adults: three-City Dijon cohort study. *BMJ* 2013;**347**:f4240.

37 Kim LG, Adamson J, Ebrahim S. Influence of life-style choices on locomotor disability, arthritis and cardiovascular disease in older women: prospective cohort study. *Age Ageing* 2013;**42**:696-701.

38 Ostbye T, Taylor DH, Jr., Krause KM*, et al.* The role of smoking and other modifiable lifestyle risk factors in maintaining and restoring lower body mobility in middle-aged and older Americans: results from the HRS and AHEAD. Health and Retirement Study. Asset and Health Dynamics Among the Oldest Old. *J Am Geriatr Soc* 2002;**50**:691-9.

39 Tas U, Verhagen AP, Bierma-Zeinstra SM*, et al.* Incidence and risk factors of disability in the elderly: the Rotterdam Study. *Prev Med* 2007;**44**:272-8.

40 Walter S, Kunst A, Mackenbach J*, et al.* Mortality and disability: the effect of overweight and obesity. *Int J Obes (Lond)* 2009;**33**:1410-8.

41 Zhang S, Tomata Y, Sugiyama K*, et al.* Body mass index and the risk of incident functional disability in elderly Japanese The OHSAKI Cohort 2006 Study. *Medicine* 2016;**95**.

42 He XXZ, Baker DW. Body mass index, physical activity, and the risk of decline in overall health and physical functioning in late middle age. *Am J Public Health* 2004;**94**:1567-73.

43 Tak E, Kuiper R, Chorus A*, et al.* Prevention of onset and progression of basic ADL disability by physical activity in community dwelling older adults: a meta-analysis. *Ageing Res Rev* 2013;**12**:329-38.
